# Prediction of guide strand of microRNAs from its sequence and secondary structure

**DOI:** 10.1186/1471-2105-10-105

**Published:** 2009-04-09

**Authors:** Firoz Ahmed, Hifzur Rahman Ansari, Gajendra PS Raghava

**Affiliations:** 1 Bioinformatics Centre, Institute of Microbial Technology, Sector 39-A, Chandigarh, India

## Abstract

**Background:**

MicroRNAs (miRNAs) are produced by the sequential processing of a long hairpin RNA transcript by Drosha and Dicer, an RNase III enzymes, and form transitory small RNA duplexes. One strand of the duplex, which incorporates into RNA-induced silencing complex (RISC) and silences the gene expression is called guide strand, or miRNA; while the other strand of duplex is degraded and called the passenger strand, or miRNA*. Predicting the guide strand of miRNA is important for better understanding the RNA interference pathways.

**Results:**

This paper describes support vector machine (SVM) models developed for predicting the guide strands of miRNAs. All models were trained and tested on a dataset consisting of 329 miRNA and 329 miRNA* pairs using five fold cross validation technique. Firstly, models were developed using mono-, di-, and tri-nucleotide composition of miRNA strands and achieved the highest accuracies of 0.588, 0.638 and 0.596 respectively. Secondly, models were developed using split nucleotide composition and achieved maximum accuracies of 0.553, 0.641 and 0.602 for mono-, di-, and tri-nucleotide respectively. Thirdly, models were developed using binary pattern and achieved the highest accuracy of 0.708. Furthermore, when integrating the secondary structure features with binary pattern, an accuracy of 0.719 was seen. Finally, hybrid models were developed by combining various features and achieved maximum accuracy of 0.799 with sensitivity 0.781 and specificity 0.818. Moreover, the performance of this model was tested on an independent dataset that achieved an accuracy of 0.80. In addition, we also compared the performance of our method with various siRNA-designing methods on miRNA and siRNA datasets.

**Conclusion:**

In this study, first time a method has been developed to predict guide miRNA strands, of miRNA duplex. This study demonstrates that guide and passenger strand of miRNA precursors can be distinguished using their nucleotide sequence and secondary structure. This method will be useful in understanding microRNA processing and can be implemented in RNA silencing technology to improve the biological and clinical research. A web server has been developed based on SVM models described in this study .

## Background

MicroRNAs (miRNAs) are ~22 nucleotide (nt) single stranded RNA molecules that are generated from endogenous hairpin like transcripts [1]. These are highly conserved molecules, which are expressed in most of the eukaryotes (e.g. animals, plants) and in viruses and help to regulate the expression of genes in a sequence specific manner [2-5]. miRNAs play very important role in maintaining the normal physiological processes such as developmental timing, differentiation, apoptosis, and genome integrity [5]. Aberrant expression of miRNAs is associated with a number of diseases including cancer [6,7]. In animals, biogenesis of miRNA involves a series of coordinated processes. The transcription of miRNA gene into a long primary transcript forms a hairpin like structure called pri-miRNA, which is processed in the nucleus by Drosha to generate hairpin precursor sequence, pre-miRNA. The pre-miRNA is transported to cytoplasm for further processing by Dicer, leading to generation of a transient intermediate of ~22 nt long imperfect duplex of miRNA:miRNA*. Subsequently, the duplex unwinds and miRNA strand is loaded into RISC [8-11]. The miRNA in RISC acts as a guide strand to find the complementary site in mRNA, thereby suppresses the translational activity of the target mRNA. The miRNA*, also known as the passenger strand, is degraded when the duplex is unwound [12].

In the recent years, RNA interference (RNAi) has become a popular tool in many applications ranging from functional studies to therapeutics. The two main classes of molecules that trigger this mechanism are miRNAs and small interfering RNAs (siRNAs). The major limitations for their applicability are that these RNAs have short half-life and off target effects. Rational design and chemical modifications of siRNAs were used in an attempt to overcome these limitations [13-24]. One of the most crucial steps in the RNAi pathway is RISC formation during which the Argonaute 2 protein (in Humans) incorporates one of the siRNA duplex strands. This strand solely determines the target for gene silencing [25,26]. Previous studies have clearly shown that the selection of one strand from miRNA/siRNA as a guide is not random and is governed by their associated features [27-29], such as low thermodynamic stability at 5'-end of the guide strand compared to the passenger strand and presence of specific nucleotide at particular positions [22,28,29]. An earlier study also indicated that the competition for the binding of siRNAs to RISC is dependent on the siRNAs potencies such as highly potent siRNAs bind to RISC effectively [17]. As most components of the miRNA and siRNA pathways are identical, models developed on miRNA can likely be applied to siRNAs and *vice-versa*. Mostly the features associated with guide strands are derived from studies of siRNAs, which are perfect complementary 19 nt long duplexes with 2 nt overhang at 3'-end. On the contrary, miRNAs form imperfect base pairs with complementary strand of miRNA* and thus result in mismatches and bulges which destabilizes the transitory duplex. These structural features of precursors have been shown to be important for efficient processing of miRNA biogenesis [30]. miRNA can be generated either from 5' or 3' arm of hairpin; however, there are reports that both arms of hairpin can generate guide strand which further complicates the understanding about the process of active RISC formation [6]. A study shows that when relative free energies of the 5'-end of both strand of a miRNA duplex is similar, no asymmetry exist and both strand equally accumulate *in-vivo *[29]. A recent study revealed that although miRNAs are more abundant than miRNA* in the biological systems, but some species of miRNA* are also reported in abundance especially in S2 cells [31]. An earlier study also showed that ineffective siRNAs can give more than 80% gene silencing activity in the S2 cells while similar siRNAs are ineffective in other cells like CHO-K1, HeLa, and E14TG2a, indicating that other than biophysical properties of duplex some unidentified factors are likely to have a significant role in the guide strand selection [22]. However, mutations in gene sequence may change the properties of miRNA to become miRNA* which may result an event where a wrong strand would integrate into RISC and could adversely affect gene regulation. The increase in number of both miRNA and miRNA* sequences detected by deep sequencing efforts necessitate the characterization of these sequences by computational methods and development of models to predict highly abundant strand, miRNA, in biological systems. Further studies in this regard become very useful for selecting and designing an effective strand to knockdown expression of a specific gene. To the best of our knowledge, computational studies have not been conducted so far to classify the miRNA and miRNA* strand.

In this study, an attempt has been made to develop computational method for discriminating miRNA and miRNA* strand for the very first time. All miRNA datasets were collected from miRBase (Release 11.0) [32]. In this study, we utilized various features of miRNA for developing prediction method by using SVM technique.

## Results

**Figure 1:**
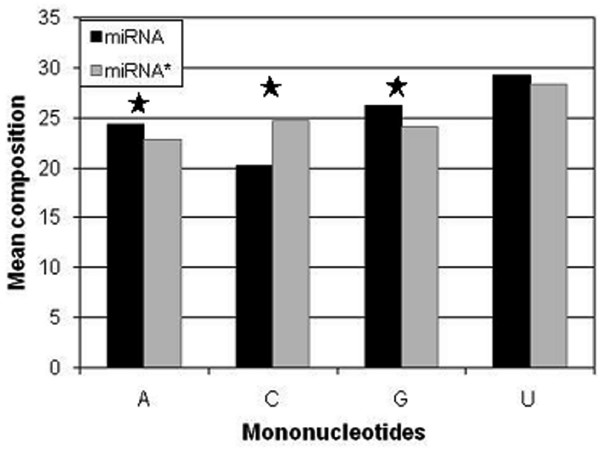
Average percent composition of four nucleotides in miRNA and miRNA* sequences. A star mark shows statistical difference in nucleotide composition of miRNA and miRNA* (p-value < 0.05).

**Figure 2:**
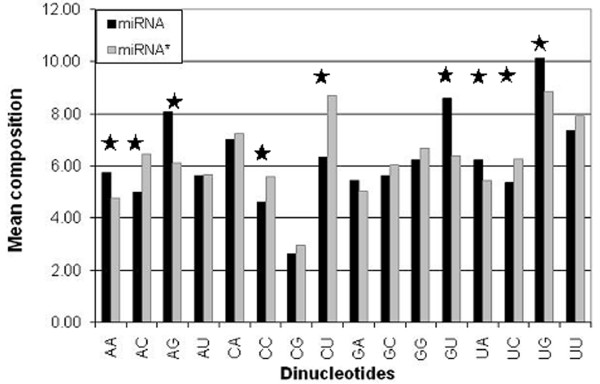
Average percent composition of each of sixteen dinucleotides in miRNA and miRNA* sequences. A star mark shows statistical difference in dinucleotides of miRNA and miRNA* (p-value < 0.05).

We computed and compared base composition (mono-, di-nucleotide) of miRNA and miRNA*, in order to understand whether they are compositionally different. Furthermore, we also examined whether difference in composition of miRNA and miRNA* is statistically significant using student's t-test (Table S1 in Additional file 1). As shown in Figure 1, bases A and G are abundant in miRNA while miRNA* is rich in base C. Figure 2 shows that dinucleotide composition is clearly different in these sequences. Dinucleotides GU, AG, UG, and AA are among the most prominent in miRNA while CU, AC, CC, and UC are prominent in miRNA*. These results clearly indicate that both stands are compositionally different, which means composition can be used to predict miRNA strand.

### Prediction using nucleotide sequence

#### Composition based SVM models

In this study, we considered miRNAs as positive example and miRNAs* as negative example. Initially, SVM models were evaluated using standard five-fold cross validation technique and achieved accuracies of 0.643, 0.725 and 0.775 using mono-, di-, and tri-nucleotide compositions respectively (Table S2a in Additional file 1). It has been observed that members of a miRNA family have high similarity, which may effect on performance of model if training and testing set having similar sequences. In order to overcome this bias, we evaluate models using non-redundant five-fold cross validation technique [33], where all member of a family were kept into one set (See "Methods"). We achieved maximum accuracies of 0.588, 0.638 and 0.596 using mono-, di-, and tri-nucleotide composition respectively (Table S2b in Additional file 1). In order to avoid biased performance, we evaluated all models in this study using non-redundant five-fold cross-validation.

#### Split nucleotide composition

In this case each sequence was divided into two equal parts, and composition of each part was calculated then added to each other to get double vector dimension. In this way, we achieved highest accuracy of 0.553, 0.641 and 0.602 for mono-, di-, and tri-nucleotide respectively (Table S3 in Additional file 1).

#### Binary pattern

Here SVM models were developed using binary pattern features, which revealed the occurrence of position specific nucleotide. In our datasets, the length of miRNA/miRNA* sequences varied from 18 nt to 26 nt were obtained from 20 different organisms (Table S4a, b in Additional file 1), whereas SVM require fixed length pattern. Hence fixed numbers of nucleotides were taken from 5'- and 3'-ends of sequences. Initially, we developed models using window size of 6 to 18 nt from 5'-end of sequence and achieved maximum accuracy 0.708 for 11 nt. Similarly, we developed models using window size of 6 to 18 nt from 3'-end (Figure S1 in Additional file 1), and achieved maximum accuracy of 0.693 for 13 nt (Table 1).

**Table 1:** Performance of various SVM models based on binary pattern developed using nucleotides from 5' and 3'-end of sequence.

**Window size**	**5'-end**				**3'-end**			
	**Sn**	**Sp**	**Ac**	**Mc**	**Sn**	**Sp**	**Ac**	**Mc**
6	0.660	0.723	0.692	0.38	0.611	0.678	0.644	0.29
7	0.693	0.684	0.688	0.38	0.660	0.654	0.657	0.31
8	0.696	0.687	0.692	0.38	0.657	0.632	0.644	0.29
9	0.678	0.699	0.688	0.38	0.647	0.657	0.652	0.30
10	0.678	0.705	0.692	0.38	0.660	0.635	0.647	0.29
**11**	**0.702**	**0.714**	**0.708**	**0.42**	0.666	0.641	0.654	0.31
12	0.690	0.693	0.692	0.38	0.644	0.657	0.651	0.30
**13**	0.669	0.708	0.688	0.38	**0.730**	**0.657**	**0.693**	**0.39**
14	0.726	0.647	0.687	0.38	0.690	0.651	0.670	0.34
15	0.647	0.723	0.685	0.37	0.629	0.696	0.663	0.33
16	0.687	0.678	0.682	0.36	0.654	0.702	0.678	0.36
17	0.693	0.669	0.681	0.36	0.663	0.678	0.670	0.34
18	0.672	0.708	0.690	0.38	0.641	0.669	0.655	0.31

Window size: number of nucleotides taken from 5'- and 3'-end of sequence

### Binary pattern and secondary structure of putative miRNA:miRNA* duplex

**Figure 3:**
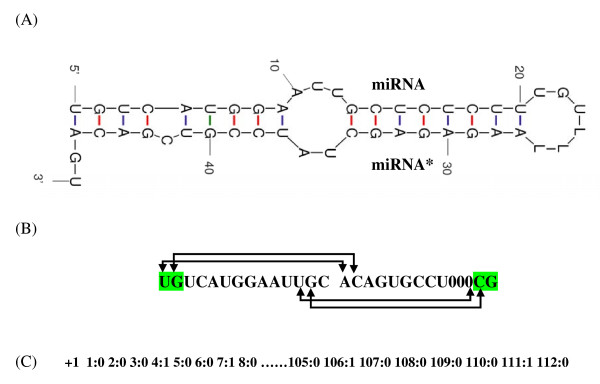
Schematic diagram of Binary pattern and secondary structure features of miRNA:miRNA* duplex. (A) Secondary structure predicted by quikfold software. (B) The sequence information of 14 nt is taken from 5'-end of miRNA and its partially complementary miRNA*. Some of the base pairs are indicated with arrows. Zero (0) indicates no base pairing occurs between complementary strands. The pattern of 14 nt + 14 nt is used to generate binary pattern. (C) Binary pattern of 112 dimensional vector is generated as input for SVM. +1 is the class for miRNA, here binary pattern is represented only for highlighted nucleotides in (B).

In previous sections, the sequence features of only single strand of miRNA were considered whereas the information of complementary strand was lacking. During the time of RISC association, the miRNA and miRNA* are present in a duplex form, therefore, we took the structural information of a strand along with its binary pattern. For this, we ligated the sequence of miRNA with their corresponding miRNA* by 3Ls to predict the secondary structure using quikfold [34,35], as described in "Methods" section (Figure 3A). This program uses the nearest-neighbor method to calculate the secondary structure. We extracted the information of secondary structure like base pairs and mismatches between the two strand and thermodynamic details from output result.

Here, we generated the models similar to the binary pattern with additional information of complementary strand. Different window size from miRNA sequences, varying from 6 nt to 18 nt from 5'-end and its base paired sequences were taken. For instance, for a 14 nt long pattern (from 5'-end of miRNA), the base pairing nucleotides present in miRNA* were also considered thus making it 28 nt (14+14). A case of mismatch, where base pairing was absent, was considered as 0 (zero) (Figure 3B). Now, the binary pattern of all nucleotides (total 28 nt), was generated (Figure 3C) giving the vector dimension 112 (28*4). This information contained both structural features as well as position specific nucleotides. Similarly binary pattern and structural feature of miRNA* was calculated as shown in Figure S2 of the Additional file 1.

Initially, we developed various models by using different window size taken from 5'- and 3'-end, and achieved a highest accuracy of 0.719 and 0.717 for 11 nt window (from 5'-end) and 6 nt window (from 3'-end) respectively (Table 2).

**Table 2:** Performance of various SVM models based on binary pattern & secondary structure feature developed using nucleotides from 5' and 3'-end of sequence.

**Window size**	**5'-end**				**3'-end**			
	**Sn**	**Sp**	**Ac**	**Mc**	**Sn**	**Sp**	**Ac**	**Mc**
**6**	0.717	0.705	0.711	0.42	**0.720**	**0.714**	**0.717**	**0.43**
7	0.708	0.687	0.698	0.40	0.720	0.708	0.714	0.43
8	0.714	0.675	0.695	0.39	0.690	0.687	0.688	0.38
9	0.714	0.696	0.705	0.41	0.696	0.684	0.690	0.38
10	0.696	0.702	0.699	0.40	0.647	0.745	0.696	0.39
**11**	**0.711**	**0.726**	**0.719**	**0.44**	0.690	0.726	0.708	0.42
12	0.699	0.699	0.699	0.40	0.699	0.711	0.705	0.41
13	0.684	0.684	0.684	0.37	0.687	0.699	0.693	0.39
14	0.690	0.681	0.685	0.37	0.705	0.730	0.717	0.43
15	0.699	0.669	0.684	0.37	0.672	0.757	0.714	0.43
16	0.717	0.638	0.678	0.36	0.669	0.754	0.711	0.42
17	0.699	0.660	0.679	0.36	0.663	0.736	0.699	0.40
18	0.687	0.663	0.675	0.35	0.675	0.696	0.685	0.37

### Hybrid models

In this case, we combined more than one types of features at a time for developing a model. In case of nucleotide composition, combinations of different types of composition were used for developing models (e.g. mono+di-nucleotide, mono+di+tri-nucleotide etc.), and achieved highest accuracy of 0.622 for mono+di-nucleotide, which remained lower than that of simple dinucleotide (Table S5 in Additional file 1). Furthermore, in case of split nucleotide composition, different combinations were also used but even then we achieved highest accuracy of 0.611 by combination of mono+di+tri-nucleotide, which is still lower than that achieved by simple dinucleotide composition (Table S6 in Additional file 1).

Previously in the binary pattern model, we only considered the information of one end of a sequence at a time, thus missing the information in the remaining part. In the hybrid models, we tried to combine the information from both ends. Firstly, equal window size from both ends was taken, ranging from 10 nt to 18 nt long and their binary patterns were generated. On five-fold cross validation, we got highest accuracy of 0.710 by combining 12 nt window from both ends (Table S7 in Additional file 1). We speculated that a combination of varying window size might improve the accuracy. Therefore, we combined the two parts in which one part has fixed window size (11 nt and 18 nt) from 5'-end, while other part has varied window size (6 nt to 18 nt) from 3'-end. Among them, we got a highest accuracy of 0.719 for the window size of 11 nt + 7 nt (5'-end + 3'-end) and accuracy of 0.710 for 18 nt +11 nt (Table S8 in Additional file 1). Similarly, we considered the 13 nt as well as 16 nt window size from 3'-end while varying pattern length from 5'-end. The highest accuracy of 0.717 was achieved with the models of 6 nt + 13 nt and 7 nt + 16 nt (Table S9 in Additional file 1). The above study clearly shows the improvement in accuracy by using features of both ends instead of one and hence reflects the significance of both ends. Therefore, the highest accuracy so far achieved is 0.719 employing *Hybrid of binary pattern *of 11 nt + 7 nt (Table S8 in Additional file 1).

Furthermore, we developed models for *Hybrid of binary pattern and secondary structure *by combining the features of binary and secondary structure of two ends of equal window size. Among all the models highest accuracy of 0.733 was achieved by using 11 nt +11 nt window (Table S10 in Additional file 1). At last, we considered one end as a fixed window (which shows highest accuracy) whereas the other end with varying window. By fixing the 5'-end we got a highest accuracy of 0.784 by using 6 nt + 6 nt and an accuracy of 0.763 by using 11 nt + 8 nt (Table 3). We also checked the similar trend by fixing 3'-end, and got highest accuracy of 0.784 by using 6 nt + 6 nt and 0.757 by using 6 nt + 14 nt (Table S11 in Additional file 1). The above result indicated that the best model to classify miRNA and miRNA* was the one developed using structural and binary features of 6 nt window size from both ends. Hence, the developed model was chosen and additional features such as G+C content, thermodynamic stability were incorporated for further studies.

**Table 3:** Performance of various hybrid SVM models based on binary pattern & secondary structure developed using fixed number N (e.g., 6, 11) of nucleotides from 5'-end and varying number of nucleotides from 3'-end of sequence.

**Window size**	**N = 6 nt (from 5'-end)**				**N = 11 nt (from 5'-end)**			
	**Sn**	**Sp**	**Ac**	**Mc**	**Sn**	**Sp**	**Ac**	**Mc**
**N+6**	**0.809**	**0.760**	**0.784**	**0.57**	0.766	0.730	0.748	0.50
N+7	0.799	0.757	0.778	0.56	0.763	0.742	0.752	0.50
**N+8**	0.802	0.742	0.772	0.55	**0.809**	**0.717**	**0.763**	**0.53**
N+9	0.781	0.754	0.768	0.54	0.787	0.711	0.749	0.50
N+10	0.772	0.748	0.760	0.52	0.748	0.708	0.728	0.46
N+11	0.745	0.781	0.763	0.53	0.739	0.726	0.733	0.47
N+12	0.751	0.754	0.752	0.50	0.766	0.690	0.728	0.46
N+13	0.766	0.751	0.758	0.52	0.754	0.717	0.736	0.47
N+14	0.781	0.733	0.757	0.51	0.736	0.714	0.725	0.45
N+15	0.806	0.711	0.758	0.52	0.742	0.714	0.728	0.46
N+16	0.778	0.739	0.758	0.52	0.739	0.726	0.733	0.47
N+17	0.781	0.726	0.754	0.51	0.760	0.705	0.733	0.47
N+18	0.742	0.751	0.746	0.49	0.766	0.687	0.726	0.45

N: Number of nucleotides from 5'-end. N+6: N is number of nucleotides from 5'-end and 6 nt from 3'-end of a sequence.

It has been elegantly demonstrated that the G+C content is one of the important features for functional siRNAs [21,36]. Hence, we tried to find out the differences between the G+C content of miRNA and miRNA* sequences. Student's t-test was employed which revealed that the G+C content in both classes is significantly different (Table S12 in Additional file 1). Therefore, we integrated the G+C features of whole sequence into the *Hybrid of binary pattern and secondary structure *models (6 nt + 6 nt). When we incorporated this information as number of G+C per 100 nt, i.e percent form, we got an accuracy of 0.748 which was lower than that of *Hybrid of binary pattern and secondary structure *model (6 nt + 6 nt). Next, we added the information as number of G+C per 1 nt and got an accuracy of 0.785 that was nearly similar to *Hybrid of binary pattern and secondary structure *model (6 nt + 6 nt). However, when we incorporated the information as number of G+C per 10 nucleotide, the accuracy increased up to 0.799 with sensitivity of 0.781, specificity of 0.818, and MCC of 0.60 (svm parameters of g = 0.01, c = 10, and j = 1). We called this last model as *Hybrid of binary pattern, secondary structure and GC*. However, we were interested to integrate the thermodynamic stability of RNA duplex to this model. We calculated the stability of 2 nt (2 window) and 3 nt (3 window) 5'-end terminal of miRNA and miRNA* by using method as described by Krol *et. al*. [27]. Student's t-test showed that 5'-end miRNAs have significantly lower stability than that of miRNAs* (Table S12 in Additional file 1). After integrating the thermodynamic feature in *Hybrid of binary pattern, secondary structure and GC *model, resulted in accuracy of 0.793 for 2 windows and 0.799 for 3 windows. This indicates that addition of thermodynamic features could not further increase the accuracy of prediction.

**Figure 4:**
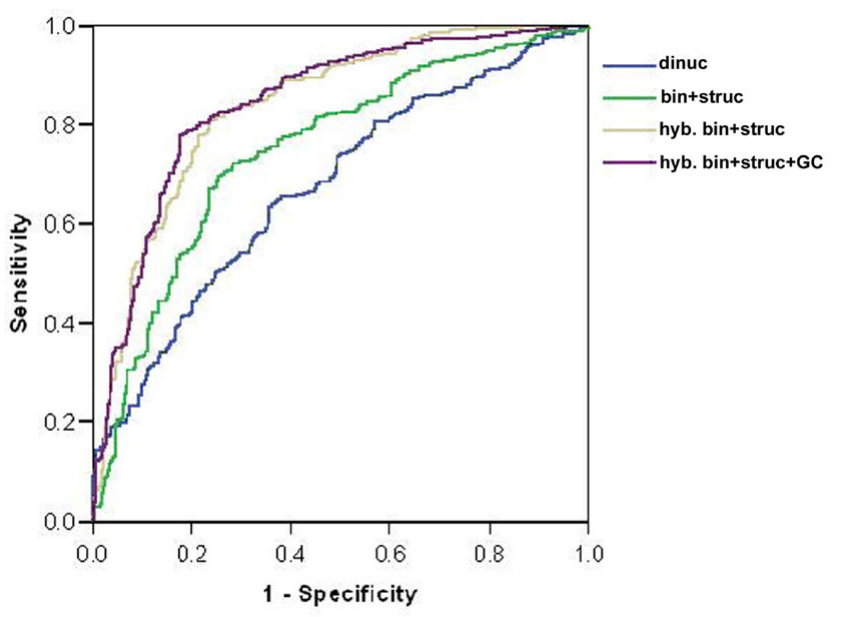
**Performance of various SVM models shown by ROC plot**. dinuc: dinucleotide composition, bin+struc: Binary pattern and secondary structure of 11 nt window from 5'-end. hyb. bin+struc: Hybrid of binary pattern and secondary structure of 6 nt window taken from both ends. hyb. bin+struc+GC: Hybrid of binary pattern, secondary structure, and GC (RISCbinder).

The performance of different models was tested by receiver operating characteristics (ROC) which plot a graph of true positive rate (sensitivity) as a function of false positive rate (1-specificity) [37]. Figure 4 shows the ROC curve on threshold independent parameters of some models. The area under the curves (AUC) of different models is: *Simple dinucleotide composition *= 0.672, *Binary pattern and secondary structure *of 11 nt pattern from 5'-end = 0.748, *Hybrid of binary pattern and secondary structure *(6 nt + 6 nt) = 0.837, *Hybrid of binary pattern, secondary structure, and GC *= 0.842. These AUC values clearly show that the two best models (*Hybrid of binary pattern and secondary structure; Hybrid of binary pattern, secondary structure, and GC*) are similar, but clearly better than the other two methods.

Though we have used non-redundant five fold cross validation, where chance of over optimization is minimum. Still overtuning of SVM parameters during the training process may result into over optimized model. Thus we also evaluate our models with five-fold cross validation using three-way data splits; where we use three sets for training; one set for validation and one set for testing. Performances of some models evaluated using above technique have been shown in Table S13a and Table S13b of the Additional file 1. Our best model, *Hybrid of binary pattern, secondary structure, and GC *(RISCbinder) achieved 0.762 accuracy, which was slightly lower than simple non-redundant five-fold cross validation (0.799). The performance might be lower due to reduction in size of training set (four-sets to three-sets).

### Performance on independent or blind dataset

**Figure 5:**
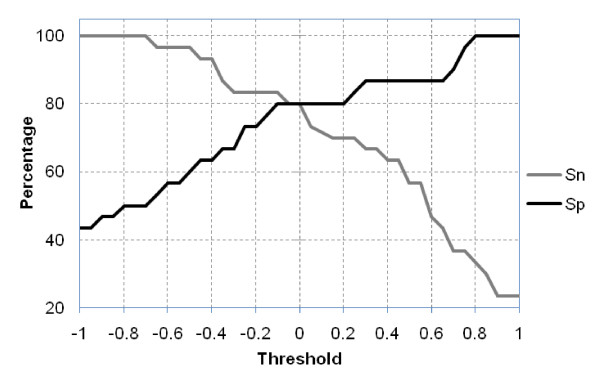
**Sensitivity (Sn) and specificity (Sp) of our method on independent dataset**.

So far the performance of developed models was assessed using five-fold cross validation. It has been shown in past that method should be evaluated on independent or blind dataset in order to make realistic evaluation [38]. Thus we assessed the performance of *Hybrid of binary pattern, secondary structure, and GC *model (RISCbinder), our best model, on independent dataset. The independent dataset contained 30 experimentally validated miRNA and its corresponding miRNA* sequences. These sequences were taken from those families, which were not used in the training datasets. Out of 30 sequences of miRNA and miRNA* (total 60), our model predicted 24 as true miRNA and 24 as true miRNA* at default threshold. That means an accuracy of 0.80 at sensitivity of 0.80 and specificity of 0.80 has been achieved (Figure 5).

### Comparison with siRNA-designing algorithms

In this study, first time models have been developed to discriminate miRNA and miRNA*. So it is difficult to compare these models with any existing method. In past numbers of methods have been developed to predict effective siRNA. Thus attempts have been made to compare our method with these siRNA-designing algorithms. These siRNA-designing algorithms can be divided into two groups: Group 1 based upon siRNA duplex terminal stability, or presence or absence of specific nucleotide at terminal position, exploited the mechanistic insight to enter an antisense strand into RISC complex for mRNA cleavage. Group 2 based upon the presence or absence of specific nucleotide at a particular position, and percentage of nucleotides in sense and antisense strand [39]. In this study we evaluate the performance of seven different algorithms: Ui-Tei [22], Amarzguioui [40], Takasaki [23], and Reynolds [21] belong to Group 1; i-Score [14], and Katoh [41] belong to Group 2. Our method is different from siRNA-designing methods in two aspects: I) our method identifies a guide strand between two strands of a duplex, whereas siRNA-designing algorithms predict the efficacy of a siRNA (i.e. regression methods) and II) our method is based on loading of guide strand to RISC, whereas siRNA-designing algorithms considered total efficacy of siRNAs from loading of antisense stand on RISC, target selection, and target cleavages [41].

We used four datasets to assess the performance: (A) 329 miRNA/miRNA* pairs used in this study, (B) 30 miRNA/miRNA* pairs used as an independent dataset, (C) 37 highly effective siRNAs and 17 ineffective siRNAs taken from Ui-Tei [22] and (D) 78 highly effective siRNAs and 39 ineffective siRNAs taken from Katoh [41].

First, we evaluate the performance of siRNA-designing algorithm on miRNA and miRNA* datasets. Here, we assign miRNA as effective siRNA and miRNA* as ineffective siRNA and evaluate performance of siRNA-designing algorithms. As shown in Figure S3–S4 of the Additional file 1 and Table 4, our method RISCbinder, performed better than other methods on both datasets (main and independent dataset of miRNA/miRNA*).

**Table 4:** Performance in form of area under curve (AUC) of our method, RISCbinder, and seven siRNA-designing algorithms on four datasets.

**Algorithm**	**329 miRNA**	**30 miRNA**	**54 siRNA**	**117 siRNA**
Ui-Tei	0.585	0.652	0.983	0.635
Amarzguioui	0.703	0.783	**1.000**	0.775
Hsieh	0.562	0.561	0.720	0.643
Takasaki	0.561	0.629	0.942	0.687
i-Score	0.577	0.815	0.992	**0.790**
Reynolds	0.451	0.578	0.900	0.654
Katoh	0.452	0.596	0.773	0.731
*RISCbinder*	***0.842***	***0.869***	*0.979*	*0.677*

In the next step, we evaluate all methods on experimentally validated siRNA datasets. We assigned an siRNA as highly effective siRNA, if it suppress gene expression more than 80% and as ineffective siRNA, if it suppress gene expression less than 20%. In case of highly effective siRNAs, we assumed that antisense strand having higher affinity for RISC than sense strand, thus considered as positive class for antisense and negative class for sense. Likewise for ineffective siRNAs, we considered that antisense strand having lowest affinity for RISC than that of sense strand and thus considered as negative for antisense and positive class for sense strand. Although there is experimental need to check the effect of ineffective siRNA targeting for both sense as well as antisense target site. Figure S5 of the Additional file 1 shows the performance of algorithms on 54 siRNA data, by ROC curve, which revealed that Amarzguioui achieved better performance followed by i-Score and Ui-Tei (Table 4). This dataset was used to derive the design rule for Ui-Tei method and also supported the finding of lower thermodynamic stability at 5'-end of guide strand than the passenger strand [28]. However our method is comparable with Ui-Tei but better than four other methods (Hsieh, Takasaki, Reynolds and Katoh). Table S14a of the Additional file 1 shows that out of 37 effective siRNA our method predicted 31 as true positive at default threshold (0). Whereas for 6 siRNAs prediction showed both sense, and antisense stand as negative but in 5 of those siRNAs, the score of antisense strand was more than the sense strand indicating that these 5 siRNAs were also predicted as functional. Table S14b of the Additional file 1 shows that our method predicted all 17 ineffective siRNAs as true negative. Therefore, the results suggested that our method on this dataset predicted the functional siRNAs with an accuracy of 0.981 with a 0.973 sensitivity and 1.00 specificity.

The evaluation on 117 siRNA data shows that, i-Score achieved better performance followed by Amarzguioui, Katoh, and Takasaki (Figure S6 in Additional file 1 and Table 4), though the same data were used for development of Katoh method. On this dataset our algorithm performed better than three other methods (Ui-Tei, Hsieh, and Reynolds). These show that our method is not only suitable for predicting miRNA, and miRNA* but also capable of predicting effective and ineffective siRNA.

## Discussion

In this study, we took the advantage of the recent miRBase, which contain the information of experimentally validated sequences of miRNA and miRNA*. Initially, we analyzed the preferences of bases in miRNA and found the prevalence of specific nucleotides within miRNA indicating that these nucleotides play important role for their RISC binding properties (Figure 1, 2). The differential nucleotide compositions prompted us to develop a suitable model, which can classify miRNA and miRNA*. Here, we developed the models by implementing the SVM and exploiting sequence and structural features associated with these RNAs. Earlier several methods have shown the composition as an important feature for the classification of biological molecules [42-45]. In the present study, we achieved a highest accuracy when dinucleotide composition was used to develop the models (Table S2b in Additional file 1). As dinucleotide offered information about neighbor nucleotide also, therefore accuracy achieved was better as compared to mononucleotide. However, with trinucleotide, we could not achieve better accuracy possibly because of small size of sequences length and lack of any conserved pattern. It has been indicated that features present at the 5'-end play important role in duplex unwinding and loading on to RISC [28]. Hence, we expected that splitting of the whole sequences into two equal parts would possibly give better discriminatory features for classification. Recently we have reported that splitting of sequence could better exploit region specific motif and hence increase the prediction accuracy [46]. However in this study split nucleotide composition did not improve the accuracy (Table S3 in Additional file 1). The relevance of position specific nucleotide associated with effective siRNAs has also been shown in the past [15,36,39]. Moreover, it has also been successfully used in other studies to classify antibacterial properties [47]. Therefore, we used binary pattern as input features to develop the model and an improved accuracy of 0.708 was noticed (Table 1). This 0.7 increase in the accuracy of binary-based model (when compared to composition based model) indicates the significance of position specific nucleotide and also supports the earlier finding [15,21,36,39,41]. The accuracy obtained here is different for different window size, which is likely to be due to impact of a particular nucleotide at specific position. The accuracy for 11 nt from 5'-end and 13 nt from 3'-end are better than other size (Table 1). In addition, the achievement of increased accuracy for 5'-end than 3'-end reflects the impact of 5'-end for discriminatory features.

Using binary pattern and secondary structure information of RNA molecules, we achieved slightly better accuracy of 0.719 at 11 nt window from 5'-end (Table 2). This is because we also incorporated the information of complementary strand along with bulges and mismatches that destabilize the structure, thereby giving natural information about the end of duplex.

Several studies have clearly shown the increase in the accuracy of prediction when combinations of numerous features were used to generate a hybrid model. However, we generated the hybrid of different compositions but couldn't achieve high accuracy. It appears that region specific nucleotides composition do not play important role in strand classification. However, when both ends were combined for binary pattern, a significant increase in accuracy was achieved (Table S7, S8, and S9 in Additional file 1). As we achieved highest accuracy for 11 nt from 5'-end and 13 nt from 3'-end, we expected an increase in accuracy upon combination of both size but did not get highest accuracy. We obtained an improved accuracy of 0.719 when a window of 11 nt from 5'-end and 7 nt from 3'-end were combined (Table S8 in Additional file 1). This is because the combination of both windows is not always additive for their accuracy, since features may depend on one another and may exhibit negative co-operativity. Similarly we achieved a highest accuracy of 0.784 by combining the binary and structural information of 6 nt window from both ends (Table 3). This indicates that the 6 nt window from both ends possibly play an important discriminatory features for SVM. We expect that by taking 12 nt stretches would possibly lead to similar sequences across different folders and hence may improve the accuracy during 5-fold cross validation. However, it was observed that despite the shorter length of sequences, the 5 fold were different from each other that ruled out the gaining of better accuracy by sequence bias. Further integration of G+C content into the binary and structural features improves the prediction accuracy up to 0.799. This was because G+C content plays an important role in determining the functionality of a strand and thus also supports earlier study [21,22,39]. The variation of accuracy by using same G+C content in different forms is noticeable. As the *Hybrid of binary pattern and secondary structure *model contains information in the form of binary pattern i.e. either 1 or 0, thus integrating the features of G+C as percent gave very high weightage and in turn diluted some background binary-structural information resulting in decrease in accuracy. However, putting G+C features in fraction form did not give any weightage with respect to binary one and thus the accuracy remained unchanged. On the other hand integrating G+C contents per 10 nt made a proper balance of weight between the features of binary-structural and G+C content and thus the accuracy increased by 0.015. This suggests that during combination of features, information should be given in proper weight. Integration of the thermodynamic stability feature at 5'-end did not increase the accuracy indicating that structural-sequence features are sufficient for classification, which is mainly determined by thermodynamics features. This study shows that a combination of different features along with specific window size play an important role in miRNA/miRNA* classification. The best model was chosen to test the efficacy of prediction on independent dataset and achieved an accuracy of 0.80 which is similar to the training sets.

In addition, we compared the performance of our method with siRNA-designing methods on different datasets. According to receiver operating characteristics (ROC), the performance of our method is best on miRNA datasets whereas most of the other methods were poor. Although our method was not trained on siRNAs datasets but has the potential to predict effective siRNAs with similar extent as Ui-Tei, and is better than Hsieh and Reynolds (Group 1 method). However, performances of these algorithms were different on different datasets because different experimental datasets were used for creating algorithms.

## Conclusion

In this study, a model has been developed to classify miRNA and miRNA* sequences with 0.799 accuracy. The model can predict which strand is in high abundance in scenarios where both miRNA and miRNA* sequences exist in the biological systems. This was accomplished by utilizing the nucleotide features associated with these sequences. Moreover, integrating the structural features of duplex RNA conferred a combined effect which gave better discriminatory features for SVM, thereby dramatically increasing the classification accuracy. The fineness of our approaches was the utilization of experimentally validated dataset of 20 diverse organisms from metazoan, plants and viruses. Though, experimental validation of the nucleotides associated with miRNA: miRNA* duplex and their influence for RISC binding is needed to understand the RNAi mechanism. Nevertheless, we expect that the algorithm developed in this method is universally applicable and will be useful to annotate the functional miRNA therefore, has a potential to improve RNAi technology.

## Methods

The study was performed on the datasets taken from miRBase version 11.0. [32]. We retrieved the sequences of miRNA* from maturestar.fa which contains total 492 sequences and its corresponding miRNAs from mature.fa. The unique sequences of miRNAs* were selected and further excluded its hairpin precursors which met the following criteria: (1) redundant or identical sequences (2) experimentally not verified miRNA or its corresponding miRNA*, (3) miRNA and miRNA* derived from the same arm, (4) either miRNA or miRNA* derived from loop region, (5) miRNA and miRNA* derived from different arms but could not form a proper duplex in hairpin structure. Finally, we got 359 sequences matured miRNA and its corresponding miRNA* i.e. 359 pairs of sequences, from 21 different organisms including viruses. These sequences were divided into two groups.

### (A) The dataset for training

The dataset comprised of 329 sequences of miRNA and its corresponding miRNA*. The investigation and development of models were carried out using features of this dataset.

### (B) The datasets for independent testing

The model, developed on training dataset was tested on independent dataset to evaluate its performance. These datasets contained 30 sequences of miRNAs and its corresponding miRNAs*. These sequences did not have homology with each other and even not with training dataset.

### The datasets of siRNAs and comparison with siRNA-designing algorithms

To access the performance of our method with other siRNA-designing algorithms, two datasets were made: (A) having 37 highly effective and 17 ineffective siRNAs taken from Ui-Tei *et. al*. [22], (B) having 78 highly effective siRNAs and 39 ineffective siRNAs taken from Katoh *et al*. [41]. To evaluate the performance of algorithms, we used i-Score designer from Ichihara *et. al*. that also integrated other methods used in our study [14]. All these methods take input of 19 nt long sequence hence, for evaluation on miRNA/miRNA* datasets, we have taken 19 nt long sequence from 5'-end. However sequences of hsa-miR-516b* and cre-miR1151a* were 18 nt long. Therefore, an additional nucleotide (U/A) was taken from their hairpin and added at 3'-end of these sequences to make them 19 nt long. For ROC comparison, Ui-Tei algorithms, a non-scoring algorithm, was converted into scoring as shown in bracket: class Ia (+3), class Ib (+2), class II (0), and class III (-3) [39]. We also changed the scoring value for class Ia, Ib, II, III to +3, +2.5, +2, and +1 respectively but got similar ROC as on previous score.

All the datasets used in this study are available in Additional file 2.

### Standard five-fold cross validation

Initially standard five-fold cross validation technique was used to evaluate the performance of models, where dataset was randomly divided into five sets. The classifier was trained on four sets and performance was assessed on the remaining fifth set. The process was repeated five times so that each set could be used once for testing. At the last, average of the five sets was calculated as the final performance.

### Non-redundant five-fold cross validation

Though our dataset is non-redundant (no two sequence are identical), still sequence may have high similarity, particularly in a miRNA family. Ideally sequence in dataset should have minimum sequence similarity (e.g., less than 30% in case of proteins) but it decrease size of dataset significantly. The performance of a SVM model is directly proportional to size of dataset used for training. In our previous study [33], we proposed non-redundant five-fold cross validation technique, where sequences in dataset were clustered based on sequence similarity. These clustered were divided into five sets; it means all sequences of a cluster were kept in one set. Thus no two sets have similar sequences; it means sequences in training and testing sets have no sequence similarity. In this study, we divided miRNA families (as described in miFam.dat of miRBase.11); into five sets it means all sequences of a family were kept into one set. Thus similar sequences were kept in one set; it means sequence similarity in training and testing sets is minimum.

### Non-redundant five-fold cross validation technique using three-way data splits

We also tested our best models using three-way data splits, where data is divided into three disjoint sets (training, validation and testing). In this technique, dataset was divided into five sets; three sets for training, one set for validating and one set for testing. Training set was used to train SVM classifier, validation set to tune the parameter of SVM classifier and test set to assess the performance of a fully-trained classifier. This process was repeated five times so that each set was used once for testing. Finally performance of method was calculated by taking average of performance on five sets. In this case a chance of over optimization was minimum as fully trained SVM classifier is only tested once on test set.

### Independent or blind dataset

It has been observed, previously studies that five-fold cross validation technique may be biased with repeat training [38]. Thus there is need to evaluate a model on an independent dataset never used for training or testing of SVM model. In this study, independent or blind dataset consists of 30 miRNA/miRNA* sequences were used. Fully trained model using non-redundant five-fold, were finally tested on independent dataset.

### Features used for models development

#### Simple nucleotide composition

Nucleotide compositions were calculated for miRNA and miRNA* sequences and have been represented by vector of different dimensions: 4 vector for mononucleotide (composition of A, C, G, and U), 16 vector for dinucleotide (composition of AA, AC, AG, CG, AU,..., UU), and 64 vector for trinucleotide (composition of AAA, AAC, AAG,..., UUU).

Please see Table S15 of the Additional file 1 for feature construction and models development which were used in this study.

#### Split nucleotide composition

In this case, we divided the whole sequence into two equal parts [46]. Nucleotide composition of each part was computed separately then composition of both parts was used to develop prediction model. In this split nucleotide composition dimension of input vector will be doubled (e.g., 8 for mononucleotide, 32 for dinucleotide composition). For instance, in case of a 22 nt long sequence, mononucleotide composition of 11 nt was taken from 5'-end and combined to mononucleotide composition of remaining 11 nt from 3'-end thereby, doubling the vector dimension 4+4 = 8.

#### Binary pattern

It represents the position specific nucleotide occurrences. In this case each nucleotide was represented by binary pattern of dimensions four (A by [1,0,0,0], C by [0,1,0,0], G by [0,0,1,0] and U by [0,0,0,1]). We also used 0 (lack of nucleotide at particular position) in secondary structure features, which is represented by [0,0,0,0]. Thus, a sequence of 14 nucleotides of miRNA was represented by a vector of dimensions 56 (4 × 14).

#### Structure of putative miRNA:miRNA* duplex

We ligated the sequence of miRNA with miRNA* using 3Ls that consider the hairpin as two separate strand when predicted the secondary structure [34,35]. In this case two types of hairpins were generated: (1) first contained miRNA at 5'-end and miRNA* at 3'-end, referred as miRNA hairpin, (2) second contained miRNA* at 5'-end and miRNA at 3'-end, referred as miRNA* hairpins. Afterwards, structures were predicted by using quikfold server (RNA 3.0) available at [35]. The output result contained .ct file, which gave structural information of putative duplex. Thermodynamic details were taken from 'loop free-energy decomposition' which were used to calculate the free energy of 2 nt and 3 nt of terminal 5'-end of miRNA as well as miRNA* sequence as calculated in [27].

### Performance Measures

The performances of models were measured using standard parameters; I) sensitivity (Sn), percent of correctly predicted miRNA; II) specificity (Sp), percent of correctly predicted miRNA*; III) accuracy (Ac), percent of correction prediction and IV) Matthews correlation coefficient (Mc). Following equations were used to calculate these parameters [48].









Where TP and FN refer to true positive and false negatives and TN and FP refer to true negatives and false positives respectively.

### Support vector machine

SVM is a kernel-based method used both for classification and regression tasks and successfully implemented in the area of biology [44,49]. SVM^light ^has been implemented in our study [50]. The software enable user to define a number of parameters, including the types of kernel (linear, polynomial, radial basis function, or sigmoid) for classification. In this study, we also used the linear and polynomial kernel but RBF kernel perform better to classify our datasets (data not shown). We optimized the SVM parameters in order to get the best performance (accuracy) on the given training dataset using five-fold cross validation. In case of linear and polynomial kernel we used default parameters. Whereas in case of RBF kernel combination of different parameters; g ∈ [0.001, 0.01, 0.1], c ∈ [1,2,3,....,10] and j ∈ [1,2,3,....,10] were used.

Statistical analysis was conducted by student's t-test using Microsoft Excel.

We used the SPSS software for ROC analysis.

## Authors' contributions

FA collected data, developed computer programs, implemented SVM, and wrote the manuscript and helped to develop the web server. HRA developed the web server and helped to improve the manuscript. GPSR conceived and coordinated the project, guided its conception and design, helped in interpretation of data, refined the drafted manuscript and gave overall supervision to the project. All authors read and approved the final manuscript.

## Supplementary Material

Additional file 1 **Supplementary Figures and Tables**. This file contains all supplementary figures and tables referred to in the main text. Click here for file

Additional file 2 **Datasets**. The compressed file contains dataset used in development of SVM models and independent datasets used for testing. Click here for file
