# Supplementary material for: Prediction of guide strand of microRNAs from its sequence and secondary structure
Source: BMC Bioinformatics. 2009 Apr 9;10:105. doi: 10.1186/1471-2105-10-105 (PMC2676257; doi:10.1186/1471-2105-10-105)
Supplement: Additional file 1 — Supplementary Figures and Tables. This file contains all supplementary figures and tables referred to in the main text. [file 1471-2105-10-105-S1.doc]

# Prediction of guide strand of microRNAs from its sequence and secondary structure

**Supplementary Figures and Tables**

Firoz Ahmed, Hifzur Rahman Ansari and Gajendra PS Raghava*

Bioinformatics Centre, Institute of Microbial Technology, Sector 39-A, Chandigarh, India.

Figures

**5’-UGUCAUGGAAUUGCUCUCUUUGU-3’**

**Figure S1:** A miRNA sequence with varying window size taken from 5’-end and 3'-end togenerate binary pattern. Here 6 nt, 7 nt, and 8 nt window size are shown by bars.

(A)


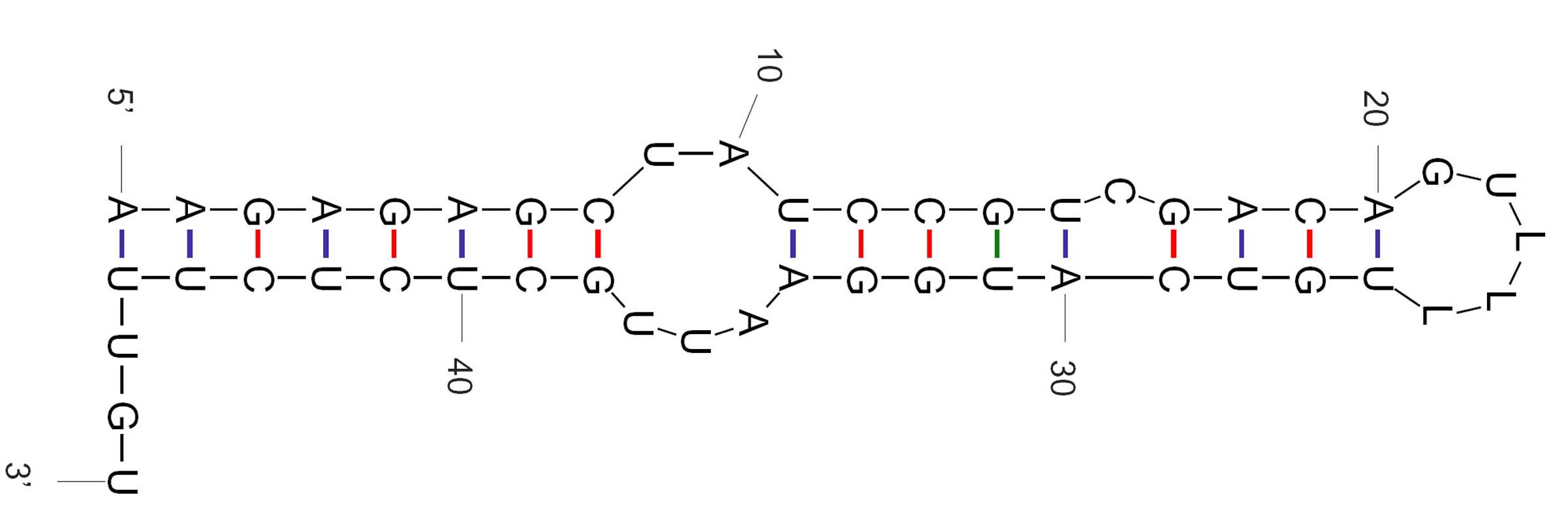


**miRNA***

**miRNA**

(B)

**AAGAGAGCUAUCCG**

**UUCUCUCG00AGGU**

(C) **-1 1:1 2:0 3:0 4:0 5:1 6:0 7:0 8:0 ……105:0 106:0 107:1 108:0 109:0 110:0 111:0 112:1**

**Figure S2**: Schematic diagram of *Binary pattern and secondary structure* features of miRNA*:miRNA duplex. (A) Secondary structure of a sequence predicted using software quikfold. (B) The sequence information of 14 nt is taken from 5’-end of miRNA* and its partially complementary miRNA. Some of the base pairs are indicated with arrows. Zero (0) indicates no base pairing occurs between complementary strands. The pattern of 14 + 14 is used to generate binary pattern. (C) Binary pattern of 112 dimensional vector is generated as input for SVM. -1 is the class for miRNA*, here binary pattern is represented only for highlighted nucleotides in (B).

(0.842)

(0.452)

(0.451)

(0.577)

(0.561)

(0.562)

(0.703)

(0.585)

**Figure S3:** Performance of our method (RISCbinder) and seven siRNA-designing algorithms on a dataset containing 329 miRNA/miRNA*. Area under curve (AUC) score is given in bracket corresponding to each method.

(0.869)

(0.596)

(0.578)

(0.815)

(0.629)

(0.561)

(0.783)

(0.652)

**Figure S4:** Performance of our method (RISCbinder) and seven siRNA-designing algorithms on an independent dataset containing 30 miRNA/miRNA*. AUC is given in bracket corresponding to each method.

(0.979)

(0.773)

(0.900)

(0.992)

(0.942)

(0.720)

(1.000)

(0.983)

**Figure S5:** Performance of our method (RISCbinder) and seven siRNA-designing algorithms on a dataset containing 54 siRNA. AUC is given in bracket corresponding to each method.

(0.677)

(0.731)

(0.654)

(0.790)

(0.687)

(0.643)

(0.775)

(0.635)

**Figure S6:** Performance of our method (RISCbinder) and seven siRNA-designing algorithms on a dataset containing 117 siRNA. AUC is given in bracket corresponding to each method.

Tables

| **Nucleotides** | | **miRNA** | **miRNA*** | **p-value** |
| --- | --- | --- | --- | --- |
| Mononucleotideeotide | A | 24.30 | 22.88 | 3.8E-02 |
|  | C | 20.24 | 24.67 | 1.1E-09 |
|  | G | 26.17 | 24.13 | 6.5E-03 |
|  | U | 29.29 | 28.32 | 2.0E-01 |
| Dinucleotide | AA | 5.75 | 4.78 | 3.0E-02 |
|  | AC | 5.00 | 6.44 | 3.3E-04 |
|  | AG | 8.07 | 6.11 | 3.4E-06 |
|  | AU | 5.62 | 5.65 | 9.4E-01 |
|  | CA | 7.02 | 7.25 | 7.7E-01 |
|  | CC | 4.62 | 5.58 | 4.2E-02 |
|  | CG | 2.63 | 2.97 | 2.9E-01 |
|  | CU | 6.32 | 8.68 | 3.7E-08 |
|  | GA | 5.46 | 5.01 | 2.4E-01 |
|  | GC | 5.62 | 6.02 | 2.8E-01 |
|  | GG | 6.24 | 6.69 | 3.9E-01 |
|  | GU | 8.57 | 6.37 | 7.1E-08 |
|  | UA | 6.23 | 5.43 | 4.8E-02 |
|  | UC | 5.36 | 6.27 | 2.5E-02 |
|  | UG | 10.13 | 8.84 | 2.6E-03 |
|  | UU | 7.35 | 7.91 | 2.9E-01 |

**Table S1:** Comparison of nucleotides and dinucleotides composition between miRNA and miRNA* sequences with their p-value.

**Table S2a:** Performance of SVM-based models developed using mono-,di- and tri-nucleotide composition; evaluated using standard five-fold cross validation.

|  | **Mononucleotide (vector=4)** | | | | **Dinucleotide (vector=16)** | | | | **Trinucleotide (vector=64)** | | | |
| --- | --- | --- | --- | --- | --- | --- | --- | --- | --- | --- | --- | --- |
| **Th^*^** | **Sn** | **Sp** | **Ac** | **Mc** | **Sn** | **Sp** | **Ac** | **Mc** | **Sn** | **Sp** | **Ac** | **Mc** |
| -1 | 0.875 | 0.255 | 0.565 | 0.17 | 1.000 | 0.015 | 0.508 | 0.09 | 0.970 | 0.237 | 0.603 | 0.30 |
| -0.9 | 0.848 | 0.368 | 0.608 | 0.25 | 1.000 | 0.033 | 0.517 | 0.13 | 0.961 | 0.280 | 0.620 | 0.33 |
| -0.8 | 0.836 | 0.395 | 0.616 | 0.26 | 1.000 | 0.070 | 0.535 | 0.19 | 0.951 | 0.347 | 0.649 | 0.37 |
| -0.7 | 0.809 | 0.426 | 0.617 | 0.25 | 0.997 | 0.119 | 0.558 | 0.24 | 0.942 | 0.380 | 0.661 | 0.39 |
| -0.6 | 0.781 | 0.456 | 0.619 | 0.25 | 0.991 | 0.192 | 0.591 | 0.30 | 0.921 | 0.438 | 0.679 | 0.41 |
| -0.5 | 0.775 | 0.486 | 0.631 | 0.27 | 0.982 | 0.264 | 0.623 | 0.35 | 0.906 | 0.492 | 0.699 | 0.44 |
| -0.4 | 0.751 | 0.532 | 0.641 | 0.29 | 0.967 | 0.350 | 0.658 | 0.40 | 0.885 | 0.550 | 0.717 | 0.46 |
| -0.3 | 0.733 | 0.553 | 0.643 | 0.29 | 0.945 | 0.420 | 0.682 | 0.43 | 0.851 | 0.626 | 0.739 | 0.49 |
| -0.2 | 0.708 | 0.590 | 0.649 | 0.30 | 0.891 | 0.495 | 0.693 | 0.42 | 0.815 | 0.675 | 0.745 | 0.49 |
| -0.1 | 0.684 | 0.617 | 0.651 | 0.30 | 0.793 | 0.623 | 0.708 | 0.42 | 0.790 | 0.745 | 0.768 | 0.54 |
| 0 | **0.657** | **0.629** | **0.643** | **0.29** | **0.696** | **0.754** | **0.725** | **0.45** | **0.760** | **0.790** | **0.775** | **0.55** |
| 0.1 | 0.605 | 0.651 | 0.628 | 0.26 | 0.611 | 0.848 | 0.730 | 0.47 | 0.739 | 0.836 | 0.787 | 0.58 |
| 0.2 | 0.581 | 0.681 | 0.631 | 0.26 | 0.559 | 0.903 | 0.731 | 0.49 | 0.714 | 0.878 | 0.796 | 0.60 |
| 0.3 | 0.556 | 0.705 | 0.631 | 0.26 | 0.526 | 0.942 | 0.734 | 0.51 | 0.669 | 0.906 | 0.787 | 0.59 |
| 0.4 | 0.532 | 0.723 | 0.628 | 0.26 | 0.486 | 0.961 | 0.723 | 0.51 | 0.644 | 0.915 | 0.780 | 0.58 |
| 0.5 | 0.514 | 0.733 | 0.623 | 0.25 | 0.465 | 0.985 | 0.725 | 0.53 | 0.611 | 0.933 | 0.772 | 0.57 |
| 0.6 | 0.495 | 0.772 | 0.634 | 0.28 | 0.444 | 0.988 | 0.716 | 0.51 | 0.547 | 0.939 | 0.743 | 0.53 |
| 0.7 | 0.462 | 0.790 | 0.626 | 0.27 | 0.426 | 0.997 | 0.711 | 0.51 | 0.517 | 0.957 | 0.737 | 0.53 |
| 0.8 | 0.447 | 0.815 | 0.631 | 0.28 | 0.401 | 0.997 | 0.699 | 0.50 | 0.471 | 0.976 | 0.723 | 0.52 |
| 0.9 | 0.416 | 0.821 | 0.619 | 0.26 | 0.383 | 1.000 | 0.692 | 0.49 | 0.441 | 0.985 | 0.713 | 0.51 |
| 1 | 0.322 | 0.857 | 0.590 | 0.21 | 0.249 | 1.000 | 0.625 | 0.38 | 0.240 | 0.985 | 0.613 | 0.34 |

Th*: Threshold, Sn: sensitivity, Sp: specificity, Ac: accuracy, Mc: Mathews correlation coefficient.

**Table S2b:** Performance of SVM-based models developed using mono-,di- and tri-nucleotide composition; evaluated using non-redundant five-fold cross validation.

|  | **Mononucleotide (vector=4)** | | | | **Dinucleotide (vector=16)** | | | | **Trinucleotide (vector=64)** | | | |
| --- | --- | --- | --- | --- | --- | --- | --- | --- | --- | --- | --- | --- |
| **Th** | **Sn** | **Sp** | **Ac** | **Mc** | **Sn** | **Sp** | **Ac** | **Mc** | **Sn** | **Sp** | **Ac** | **Mc** |
| -1 | 0.930 | 0.140 | 0.535 | 0.11 | 0.909 | 0.210 | 0.559 | 0.17 | 0.961 | 0.134 | 0.547 | 0.17 |
| -0.9 | 0.857 | 0.185 | 0.521 | 0.06 | 0.888 | 0.237 | 0.562 | 0.16 | 0.954 | 0.164 | 0.559 | 0.19 |
| -0.8 | 0.839 | 0.219 | 0.529 | 0.07 | 0.863 | 0.295 | 0.579 | 0.19 | 0.927 | 0.182 | 0.555 | 0.16 |
| -0.7 | 0.806 | 0.280 | 0.543 | 0.10 | 0.854 | 0.340 | 0.597 | 0.23 | 0.891 | 0.216 | 0.553 | 0.14 |
| -0.6 | 0.766 | 0.337 | 0.552 | 0.11 | 0.821 | 0.383 | 0.602 | 0.23 | 0.851 | 0.264 | 0.558 | 0.14 |
| -0.5 | 0.714 | 0.423 | 0.568 | 0.14 | 0.802 | 0.432 | 0.617 | 0.25 | 0.806 | 0.328 | 0.567 | 0.15 |
| -0.4 | 0.672 | 0.471 | 0.571 | 0.15 | 0.760 | 0.462 | 0.611 | 0.23 | 0.772 | 0.380 | 0.576 | 0.17 |
| -0.3 | 0.638 | 0.511 | 0.575 | 0.15 | 0.730 | 0.508 | 0.619 | 0.24 | 0.708 | 0.429 | 0.568 | 0.14 |
| -0.2 | 0.605 | 0.544 | 0.575 | 0.15 | 0.666 | 0.556 | 0.611 | 0.22 | 0.669 | 0.495 | 0.582 | 0.17 |
| -0.1 | **0.556** | **0.620** | **0.588** | **0.18** | **0.657** | **0.620** | **0.638** | **0.28** | 0.620 | 0.553 | 0.587 | 0.17 |
| 0 | 0.514 | 0.672 | 0.593 | 0.19 | 0.581 | 0.660 | 0.620 | 0.24 | **0.571** | **0.620** | **0.596** | **0.19** |
| 0.1 | 0.495 | 0.708 | 0.602 | 0.21 | 0.541 | 0.687 | 0.614 | 0.23 | 0.474 | 0.690 | 0.582 | 0.17 |
| 0.2 | 0.432 | 0.733 | 0.582 | 0.17 | 0.508 | 0.733 | 0.620 | 0.25 | 0.447 | 0.739 | 0.593 | 0.19 |
| 0.3 | 0.395 | 0.775 | 0.585 | 0.18 | 0.465 | 0.778 | 0.622 | 0.26 | 0.374 | 0.772 | 0.573 | 0.16 |
| 0.4 | 0.374 | 0.812 | 0.593 | 0.21 | 0.426 | 0.799 | 0.613 | 0.24 | 0.313 | 0.809 | 0.561 | 0.14 |
| 0.5 | 0.356 | 0.848 | 0.602 | 0.23 | 0.365 | 0.833 | 0.599 | 0.22 | 0.258 | 0.839 | 0.549 | 0.12 |
| 0.6 | 0.295 | 0.888 | 0.591 | 0.23 | 0.337 | 0.863 | 0.600 | 0.24 | 0.213 | 0.875 | 0.544 | 0.12 |
| 0.7 | 0.268 | 0.903 | 0.585 | 0.22 | 0.313 | 0.878 | 0.596 | 0.23 | 0.149 | 0.900 | 0.524 | 0.07 |
| 0.8 | 0.222 | 0.921 | 0.571 | 0.20 | 0.258 | 0.903 | 0.581 | 0.21 | 0.109 | 0.930 | 0.520 | 0.07 |
| 0.9 | 0.201 | 0.933 | 0.567 | 0.20 | 0.207 | 0.933 | 0.570 | 0.20 | 0.082 | 0.942 | 0.512 | 0.05 |
| 1 | 0.155 | 0.948 | 0.552 | 0.17 | 0.188 | 0.954 | 0.571 | 0.22 | 0.073 | 0.951 | 0.512 | 0.05 |

**Table S3:** Performance of SVM-based models developed using split nucleotide composition (mono-,di-, and tri-nucleotide).

|  | **Mononucleotide (vector=8)** | | | | **Dinucleotide (vector=32)** | | | | **Trinucleotide (vector=128)** | | | |
| --- | --- | --- | --- | --- | --- | --- | --- | --- | --- | --- | --- | --- |
| **Th** | **Sn** | **Sp** | **Ac** | **Mc** | **Sn** | **Sp** | **Ac** | **Mc** | **Sn** | **Sp** | **Ac** | **Mc** |
| -1 | 0.882 | 0.143 | 0.512 | 0.04 | 0.994 | 0.015 | 0.505 | 0.04 | 1.000 | 0.000 | 0.500 | 0 |
| -0.9 | 0.854 | 0.198 | 0.526 | 0.07 | 0.988 | 0.030 | 0.509 | 0.06 | 1.000 | 0.000 | 0.500 | 0 |
| -0.8 | 0.802 | 0.228 | 0.515 | 0.04 | 0.967 | 0.055 | 0.511 | 0.05 | 1.000 | 0.000 | 0.500 | 0 |
| -0.7 | 0.754 | 0.277 | 0.515 | 0.03 | 0.954 | 0.091 | 0.523 | 0.09 | 1.000 | 0.000 | 0.500 | 0 |
| -0.6 | 0.714 | 0.347 | 0.530 | 0.07 | 0.903 | 0.152 | 0.527 | 0.08 | 1.000 | 0.003 | 0.502 | 0.04 |
| -0.5 | 0.687 | 0.395 | 0.541 | 0.09 | 0.851 | 0.237 | 0.544 | 0.11 | 1.000 | 0.018 | 0.509 | 0.10 |
| -0.4 | 0.663 | 0.435 | 0.549 | 0.10 | 0.802 | 0.325 | 0.564 | 0.15 | 0.973 | 0.079 | 0.526 | 0.12 |
| -0.3 | 0.629 | 0.486 | 0.558 | 0.12 | 0.748 | 0.447 | 0.597 | 0.20 | 0.906 | 0.167 | 0.537 | 0.11 |
| -0.2 | 0.587 | 0.526 | 0.556 | 0.11 | 0.699 | 0.535 | 0.617 | 0.24 | 0.787 | 0.331 | 0.559 | 0.13 |
| -0.1 | **0.544** | **0.562** | **0.553** | **0.11** | **0.614** | **0.669** | **0.641** | **0.28** | **0.635** | **0.568** | **0.602** | **0.20** |
| 0 | 0.489 | 0.611 | 0.550 | 0.10 | 0.547 | 0.742 | 0.644 | 0.29 | 0.392 | 0.772 | 0.582 | 0.18 |
| 0.1 | 0.444 | 0.651 | 0.547 | 0.10 | 0.416 | 0.790 | 0.603 | 0.22 | 0.237 | 0.915 | 0.576 | 0.21 |
| 0.2 | 0.401 | 0.696 | 0.549 | 0.10 | 0.319 | 0.833 | 0.576 | 0.18 | 0.134 | 0.961 | 0.547 | 0.17 |
| 0.3 | 0.389 | 0.736 | 0.562 | 0.13 | 0.252 | 0.894 | 0.573 | 0.19 | 0.055 | 0.994 | 0.524 | 0.14 |
| 0.4 | 0.365 | 0.793 | 0.579 | 0.17 | 0.195 | 0.933 | 0.564 | 0.19 | 0.021 | 1.000 | 0.511 | 0.10 |
| 0.5 | 0.319 | 0.815 | 0.567 | 0.15 | 0.170 | 0.954 | 0.562 | 0.20 | 0.021 | 1.000 | 0.511 | 0.10 |
| 0.6 | 0.271 | 0.842 | 0.556 | 0.14 | 0.100 | 0.976 | 0.538 | 0.16 | 0.009 | 1.000 | 0.505 | 0.07 |
| 0.7 | 0.237 | 0.857 | 0.547 | 0.12 | 0.070 | 0.982 | 0.526 | 0.13 | 0.003 | 1.000 | 0.502 | 0.04 |
| 0.8 | 0.195 | 0.888 | 0.541 | 0.11 | 0.043 | 0.991 | 0.517 | 0.11 | 0.000 | 1.000 | 0.500 | 0 |
| 0.9 | 0.167 | 0.897 | 0.532 | 0.09 | 0.024 | 1.000 | 0.512 | 0.11 | 0.000 | 1.000 | 0.500 | 0 |
| 1 | 0.143 | 0.915 | 0.529 | 0.09 | 0.003 | 1.000 | 0.502 | 0.04 | 0.000 | 1.000 | 0.500 | 0 |

**Table S4a**: Organism-wise distribution of miRNA:miRNA* pairs.

| **Organisms** | | **Name** | **No. of miRNA** |
| --- | --- | --- | --- |
| **Protozoa** | B. taurus | bta | 2 |
|  | C. elegans | cel | 1 |
|  | D. melanogaster | dme | 5 |
|  | D. rerio | dre | 19 |
|  | G. gallus | gga | 2 |
|  | H. sapiens | hsa | 154 |
|  | M. mulata | mml | 1 |
|  | M. musculus | mmu | 71 |
|  | R. norvegicus | rno | 15 |
|  | S. mediterranea | sme | 10 |
| **Protista** | C. reinhardtii | cre | 12 |
| **Plants** | A. thaliana | ath | 3 |
|  | P. patens | ppt | 4 |
| **Viruses** | Epstein Barr virus | ebv | 6 |
|  | Human cytomegalo virus | hcmv | 2 |
|  | Kaposi sarcoma-associated  Herpes virus | kshv | 2 |
|  | Mouse cytomegalo virus | mcmv | 5 |
|  | Mareks disease virus | mdv1 | 7 |
|  | Mareks disease virus type 2 | mdv2 | 5 |
|  | Rhesus monkey rhadino virus | rrv | 3 |

**Table S4b**: Length-wise distribution of miRNA and miRNA* sequences.

| **Length** | **miRNA** | **miRNA*** |
| --- | --- | --- |
| 18 | 0 | 2 |
| 19 | 3 | 4 |
| 20 | 7 | 14 |
| 21 | 59 | 37 |
| 22 | 159 | 250 |
| 23 | 93 | 15 |
| 24 | 7 | 3 |
| 25 | 1 | 3 |
| 26 | 0 | 1 |
| **Total** | **329** | **329** |

**Table S5:** Performance of hybrid SVM models developed using various combination of nucleotide composition*.*

| **Composition** | **Vector** | **Sn** | **Sp** | **Ac** | **Mc** | **g** | **c** | **j** | **Th** |
| --- | --- | --- | --- | --- | --- | --- | --- | --- | --- |
| **Mono+di** | **20** | **0.617** | **0.626** | **0.622** | **0.24** | **0.001** | **2** | **1** | **-0.1** |
| Mono+tri | 68 | 0.593 | 0.635 | 0.614 | 0.23 | 0.001 | 5 | 2 | 0 |
| Di+tri | 80 | 0.620 | 0.596 | 0.608 | 0.22 | 0.001 | 3 | 1 | -0.1 |
| Mono+di+tri | 84 | 0.593 | 0.629 | 0.611 | 0.22 | 0.001 | 1 | 1 | -0.1 |

Mono: Mononucleotide, Di: Dinucleotide, Tri: Trinucleotide,

**Table S6:** Performance of hybrid SVM models developed using various combination of split nucleotide composition.

| **Composition** | **Vector** | **Sn** | **Sp** | **Ac** | **Mc** | **g** | **c** | **j** | **Th** |
| --- | --- | --- | --- | --- | --- | --- | --- | --- | --- |
| Mono+di | 40 | 0.599 | 0.571 | 0.585 | 0.17 | 0.001 | 2 | 1 | -0.1 |
| Mono+tri | 136 | 0.596 | 0.620 | 0.608 | 0.22 | 0.001 | 1 | 2 | -0.1 |
| Di+tri | 160 | 0.526 | 0.690 | 0.608 | 0.22 | 0.001 | 1 | 2 | -0.1 |
| **Mono+di+tri** | **168** | **0.450** | **0.772** | **0.611** | **0.23** | **0.001** | **1** | **2** | **-0.1** |

**Table S7:** Performance of hybrid SVM models developed using various combination of binary pattern feature developed using nucleotides from 5’ and 3’-end of sequence.

| **Window size** | **Sn** | **Sp** | **Ac** | **Mc** | **g** | **c** | **j** | **Th** |
| --- | --- | --- | --- | --- | --- | --- | --- | --- |
| 10+10 | 0.684 | 0.714 | 0.699 | 0.40 | 0.001 | 3 | 1 | -0.1 |
| 11+11 | 0.690 | 0.717 | 0.704 | 0.41 | 0.01 | 1 | 1 | -0.1 |
| **12+12** | **0.726** | **0.693** | **0.710** | **0.42** | **0.001** | **4** | **1** | **-0.1** |
| 13+13 | 0.681 | 0.717 | 0.699 | 0.40 | 0.1 | 2 | 1 | -0.1 |
| 14+14 | 0.693 | 0.693 | 0.693 | 0.39 | 0.001 | 6 | 1 | -0.1 |
| 15+15 | 0.690 | 0.708 | 0.699 | 0.40 | 0.1 | 1 | 2 | -0.1 |
| 16+16 | 0.723 | 0.678 | 0.701 | 0.40 | 0.01 | 10 | 2 | -0.1 |
| 17+17 | 0.723 | 0.693 | 0.708 | 0.42 | 0.001 | 4 | 1 | -0.1 |
| 18+18 | 0.687 | 0.720 | 0.704 | 0.41 | 0.01 | 3 | 1 | -0.1 |

Window size 10+10: denotes two window sizes, 10 nt from 5’-end and 10 nt from 3’-end of a sequence, to make a 20 nt long hybrid pattern.

**Table S8:** Performance of various hybrid SVM models based on binary pattern, where length of sequence obtained from 5’-end was fixed (e.g., 11, 18) and length of sequence obtained from 3’-end varied from 6 to 18 nucleotide.

| Window | **N=11 nt (from 5’-end)** | | | | | | | | **N=18 nt (from 5’-end)** | | | | | | | |
| --- | --- | --- | --- | --- | --- | --- | --- | --- | --- | --- | --- | --- | --- | --- | --- | --- |
| **size** | **Sn** | **Sp** | **Ac** | **Mc** | **g** | **c** | **j** | **Th** | **Sn** | **Sp** | **Ac** | **Mc** | **g** | **c** | **j** | **Th** |
| N+6 | 0.723 | 0.693 | 0.708 | 0.42 | 0.001 | 3 | 1 | -0.1 | 0.693 | 0.720 | 0.707 | 0.41 | 0.01 | 6 | 1 | -0.1 |
| **N+7** | **0.672** | **0.766** | **0.719** | **0.44** | **0.001** | **5** | **1** | **0** | 0.699 | 0.699 | 0.699 | 0.40 | 0.001 | 6 | 1 | -0.1 |
| N+8 | 0.717 | 0.705 | 0.711 | 0.42 | 0.001 | 2 | 1 | -0.1 | 0.720 | 0.687 | 0.704 | 0.41 | 0.01 | 2 | 1 | -0.2 |
| N+9 | 0.696 | 0.726 | 0.711 | 0.42 | 0.01 | 1 | 1 | -0.1 | 0.687 | 0.714 | 0.701 | 0.40 | 0.01 | 1 | 1 | -0.1 |
| N+10 | 0.714 | 0.699 | 0.707 | 0.41 | 0.001 | 2 | 1 | -0.1 | 0.726 | 0.678 | 0.702 | 0.40 | 0.01 | 7 | 1 | -0.2 |
| **N+11** | 0.690 | 0.717 | 0.704 | 0.41 | 0.01 | 1 | 1 | -0.1 | **0.723** | **0.696** | **0.710** | **0.42** | **0.01** | **2** | **1** | **-0.2** |
| N+12 | 0.684 | 0.711 | 0.698 | 0.40 | 0.001 | 6 | 1 | -0.1 | 0.723 | 0.678 | 0.701 | 0.40 | 0.01 | 5 | 1 | -0.2 |
| N+13 | 0.730 | 0.684 | 0.707 | 0.41 | 0.1 | 1 | 2 | -0.1 | 0.742 | 0.669 | 0.705 | 0.41 | 0.01 | 2 | 1 | -0.2 |
| N+14 | 0.678 | 0.717 | 0.698 | 0.40 | 0.1 | 2 | 1 | -0.1 | 0.742 | 0.672 | 0.707 | 0.41 | 0.01 | 2 | 1 | -0.2 |
| N+15 | 0.678 | 0.717 | 0.698 | 0.40 | 0.01 | 1 | 1 | -0.1 | 0.745 | 0.669 | 0.707 | 0.41 | 0.01 | 2 | 1 | -0.2 |
| N+16 | 0.730 | 0.684 | 0.707 | 0.41 | 0.01 | 10 | 3 | 0 | 0.678 | 0.723 | 0.701 | 0.40 | 0.01 | 2 | 1 | -0.1 |
| N+17 | 0.723 | 0.705 | 0.714 | 0.43 | 0.001 | 5 | 1 | -0.1 | 0.699 | 0.720 | 0.710 | 0.42 | 0.01 | 1 | 1 | -0.1 |
| N+18 | 0.717 | 0.717 | 0.717 | 0.43 | 0.001 | 5 | 1 | -0.1 | 0.687 | 0.720 | 0.704 | 0.41 | 0.01 | 3 | 1 | -0.1 |

**Table S9:** Performance of various hybrid SVM models based on binary pattern, where length of sequence obtained from 3’-end was fixed (e.g., 13, 16) and length of sequence obtained from 5’-end varied from 6 to 18 nucleotide*.*

| Window | **N=13 nt (from 3’-end)** | | | | | | | | **N=16 nt (from 3’-end)** | | | | | | | |
| --- | --- | --- | --- | --- | --- | --- | --- | --- | --- | --- | --- | --- | --- | --- | --- | --- |
| **size** | **Sn** | **Sp** | **Ac** | **Mc** | **g** | **c** | **j** | **Th** | **Sn** | **Sp** | **Ac** | **Mc** | **g** | **c** | **j** | **Th** |
| **6+N** | **0.717** | **0.717** | **0.717** | **0.43** | **0.001** | **4** | **1** | **-0.1** | 0.760 | 0.690 | 0.725 | 0.45 | 0.1 | 1 | 4 | -0.1 |
| **7+N** | 0.711 | 0.708 | 0.710 | 0.42 | 0.001 | 4 | 1 | -0.1 | **0.720** | **0.714** | **0.717** | **0.43** | **0.001** | **3** | **1** | **-0.1** |
| 8+N | 0.666 | 0.754 | 0.710 | 0.42 | 0.1 | 1 | 1 | -0.1 | 0.666 | 0.760 | 0.713 | 0.43 | 0.01 | 4 | 1 | -0.1 |
| 9+N | 0.751 | 0.666 | 0.708 | 0.42 | 0.1 | 1 | 1 | -0.2 | 0.702 | 0.708 | 0.705 | 0.41 | 0.001 | 4 | 1 | -0.1 |
| 10+N | 0.702 | 0.699 | 0.701 | 0.40 | 0.001 | 4 | 1 | -0.1 | 0.702 | 0.711 | 0.707 | 0.41 | 0.001 | 5 | 1 | -0.1 |
| 11+N | 0.730 | 0.684 | 0.707 | 0.41 | 0.1 | 1 | 2 | -0.1 | 0.702 | 0.705 | 0.704 | 0.41 | 0.001 | 5 | 1 | -0.1 |
| 12+N | 0.726 | 0.690 | 0.708 | 0.42 | 0.1 | 1 | 2 | -0.1 | 0.717 | 0.714 | 0.716 | 0.43 | 0.001 | 5 | 1 | -0.1 |
| 13+N | 0.681 | 0.717 | 0.699 | 0.40 | 0.1 | 2 | 1 | -0.1 | 0.699 | 0.705 | 0.702 | 0.40 | 0.001 | 5 | 1 | -0.1 |
| 14+N | 0.663 | 0.736 | 0.699 | 0.40 | 0.1 | 4 | 1 | -0.1 | 0.751 | 0.654 | 0.702 | 0.41 | 0.01 | 1 | 1 | -0.2 |
| 15+N | 0.702 | 0.720 | 0.711 | 0.42 | 0.01 | 1 | 1 | -0.1 | 0.693 | 0.708 | 0.701 | 0.40 | 0.1 | 1 | 3 | -0.1 |
| 16+N | 0.705 | 0.708 | 0.707 | 0.41 | 0.1 | 1 | 2 | -0.1 | 0.723 | 0.678 | 0.701 | 0.40 | 0.01 | 10 | 2 | -0.1 |
| 17+N | 0.684 | 0.745 | 0.714 | 0.43 | 0.1 | 3 | 2 | -0.1 | 0.742 | 0.657 | 0.699 | 0.40 | 0.01 | 1 | 1 | -0.2 |
| 18+N | 0.742 | 0.669 | 0.705 | 0.41 | 0.01 | 2 | 1 | -0.2 | 0.678 | 0.723 | 0.701 | 0.40 | 0.01 | 2 | 1 | -0.1 |

**Table S10:** Performance of various hybrid SVM models based on binary pattern, where length of sequence obtained from 5’-end and 3’-end varied.

| **Window**  **size** | **Sn** | Sp | **Ac** | **Mc** | **g** | **c** | **j** | **Th** |
| --- | --- | --- | --- | --- | --- | --- | --- | --- |
| 10+10 | 0.760 | 0.699 | 0.730 | 0.46 | 0.01 | 2 | 2 | 0.2 |
| **11+11** | **0.739** | **0.726** | **0.733** | **0.47** | **0.01** | **2** | **2** | **0.2** |
| 12+12 | 0.733 | 0.705 | 0.719 | 0.44 | 0.01 | 2 | 1 | -0.1 |
| 13+13 | 0.766 | 0.681 | 0.723 | 0.45 | 0.01 | 1 | 2 | 0.2 |
| 14+14 | 0.757 | 0.681 | 0.719 | 0.44 | 0.01 | 1 | 2 | 0.2 |
| 15+15 | 0.720 | 0.708 | 0.714 | 0.43 | 0.01 | 1 | 1 | -0.1 |
| 16+16 | 0.726 | 0.708 | 0.717 | 0.43 | 0.01 | 1 | 1 | -0.1 |
| 17+17 | 0.751 | 0.714 | 0.733 | 0.47 | 0.01 | 5 | 1 | -0.1 |
| 18+18 | 0.714 | 0.730 | 0.722 | 0.44 | 0.01 | 2 | 1 | -0.1 |

**Table S11:** Performance of various hybrid SVM models based on binary pattern & secondary structure, where length of sequence obtained from 3’-end was fixed (e.g., 6, 14) and length of sequence obtained from 5’-end varied from 6 to 18 nucleotide.

.

| Window | **N=6 nt (from 3’-end)** | | | | | | | | **N=14 nt (from 3’-end)** | | | | | | | |
| --- | --- | --- | --- | --- | --- | --- | --- | --- | --- | --- | --- | --- | --- | --- | --- | --- |
| **size** | **Sn** | **Sp** | **Ac** | **Mc** | **g** | **c** | **j** | **Th** | **Sn** | **Sp** | **Ac** | **Mc** | **g** | **c** | **j** | **Th** |
| **6+N** | **0.809** | **0.760** | **0.784** | **0.57** | **0.01** | **6** | **2** | **0.2** | **0.781** | **0.733** | **0.757** | **0.51** | **0.01** | **4** | **4** | **0.1** |
| 7+N | 0.769 | 0.784 | 0.777 | 0.55 | 0.01 | 5 | 2 | 0.3 | 0.757 | 0.742 | 0.749 | 0.50 | 0.01 | 2 | 1 | 0 |
| 8+N | 0.093 | 0.720 | 0.757 | 0.52 | 0.01 | 8 | 2 | 0.1 | 0.787 | 0.690 | 0.739 | 0.48 | 0.01 | 3 | 5 | 0.1 |
| 9+N | 0.766 | 0.745 | 0.755 | 0.51 | 0.01 | 7 | 5 | 0.2 | 0.754 | 0.708 | 0.731 | 0.46 | 0.01 | 3 | 5 | 0.1 |
| 10+N | 0.778 | 0.726 | 0.752 | 0.51 | 0.01 | 5 | 3 | 0.2 | 0.760 | 0.708 | 0.734 | 0.47 | 0.01 | 2 | 10 | 0.2 |
| 11+N | 0.799 | 0.708 | 0.754 | 0.51 | 0.01 | 6 | 1 | -0.2 | 0.736 | 0.714 | 0.725 | 0.45 | 0.01 | 2 | 3 | 0.2 |
| 12+N | 0.751 | 0.739 | 0.745 | 0.49 | 0.01 | 5 | 1 | -0.1 | 0.754 | 0.708 | 0.731 | 0.46 | 0.001 | 8 | 1 | -0.1 |
| 13+N | 0.745 | 0.723 | 0.734 | 0.47 | 0.01 | 4 | 1 | -0.1 | 0.726 | 0.699 | 0.713 | 0.43 | 0.001 | 9 | 1 | -0.1 |
| 14+N | 0.775 | 0.699 | 0.737 | 0.48 | 0.001 | 9 | 2 | 0.3 | 0.757 | 0.681 | 0.719 | 0.44 | 0.01 | 1 | 2 | 0.2 |
| 15+N | 0.736 | 0.720 | 0.728 | 0.46 | 0.01 | 3 | 1 | -0.1 | 0.736 | 0.687 | 0.711 | 0.42 | 0.001 | 10 | 1 | -0.1 |
| 16+N | 0.720 | 0.763 | 0.742 | 0.48 | 0.01 | 5 | 2 | 0.1 | 0.730 | 0.687 | 0.708 | 0.42 | 0.001 | 9 | 1 | -0.1 |
| 17+N | 0.693 | 0.790 | 0.742 | 0.49 | 0.01 | 7 | 1 | 0 | 0.766 | 0.672 | 0.719 | 0.44 | 0.01 | 2 | 2 | 0 |
| 18+N | 0.736 | 0.736 | 0.736 | 0.47 | 0.01 | 5 | 1 | -0.1 | 0.714 | 0.714 | 0.714 | 0.43 | 0.01 | 5 | 2 | -0.1 |

**Table S12:** Comparison of features associated with miRNA and miRNA*. Delta G: thermodynamic stability at 5’-end of putative duplex sequence for 2 (2 window), and 3 (3 window) terminal nucleotide.

| **Features** | **Mean of miRNA** | **Mean of miRNA*** | **p-value** |
| --- | --- | --- | --- |
| G+C | 46.41 | 48.80 | 1.1E-3 |
| Delta G (2) | -1.16 | -1.35 | 2.9E-5 |
| Delta G (3) | -1.45 | -1.58 | 1.3E-3 |

**Table S13a**: Performance of various SVM models based on binary pattern; evaluated by non-redundant five-fold cross validation technique using three-way data splits.

| **Window**  **size** | **validation** | | | | **test** | | | | | | | |
| --- | --- | --- | --- | --- | --- | --- | --- | --- | --- | --- | --- | --- |
|  | **Sn** | **Sp** | **Ac** | **Mc** | **Sn** | **Sp** | **Ac** | **Mc** | **g** | **c** | **j** | **Th** |
| 10+0 | 0.739 | 0.644 | 0.692 | 0.38 | 0.699 | 0.647 | 0.673 | 0.35 | 0.1 | 1 | 1 | -0.2 |
| 11+0 | 0.635 | 0.720 | 0.678 | 0.36 | 0.669 | 0.690 | 0.679 | 0.36 | 0.001 | 4 | 1 | 0 |
| 12+0 | 0.748 | 0.617 | 0.682 | 0.37 | 0.723 | 0.611 | 0.667 | 0.34 | 0.01 | 1 | 1 | -0.2 |
| 0+12 | 0.635 | 0.605 | 0.620 | 0.24 | 0.617 | 0.614 | 0.616 | 0.23 | 0.1 | 1 | 1 | -0.1 |
| 0+13 | 0.654 | 0.626 | 0.640 | 0.28 | 0.660 | 0.617 | 0.638 | 0.28 | 0.01 | 10 | 1 | -0.1 |
| 0+14 | 0.647 | 0.623 | 0.635 | 0.27 | 0.641 | 0.626 | 0.634 | 0.27 | 0.01 | 7 | 1 | -0.1 |
| 12+12 | 0.739 | 0.638 | 0.688 | 0.38 | 0.739 | 0.638 | 0.688 | 0.38 | 0.001 | 9 | 1 | -0.2 |
| 11+6 | 0.730 | 0.681 | 0.705 | 0.41 | 0.708 | 0.657 | 0.682 | 0.37 | 0.001 | 3 | 1 | -0.1 |
| 11+7 | 0.696 | 0.699 | 0.698 | 0.40 | 0.693 | 0.696 | 0.695 | 0.39 | 0.01 | 3 | 1 | -0.1 |
| 18+11 | 0.699 | 0.669 | 0.684 | 0.37 | 0.663 | 0.693 | 0.678 | 0.36 | 0.01 | 5 | 1 | -0.1 |
| 18+12 | 0.620 | 0.748 | 0.684 | 0.37 | 0.626 | 0.739 | 0.682 | 0.37 | 0.01 | 2 | 1 | 0 |
| 6+13 | 0.693 | 0.708 | 0.701 | 0.40 | 0.690 | 0.714 | 0.702 | 0.40 | 0.01 | 4 | 1 | -0.1 |
| 7+13 | 0.696 | 0.708 | 0.702 | 0.40 | 0.678 | 0.708 | 0.693 | 0.39 | 0.01 | 4 | 1 | -0.1 |
| 6+16 | 0.663 | 0.739 | 0.701 | 0.40 | 0.663 | 0.745 | 0.704 | 0.41 | 0.001 | 5 | 1 | 0 |
| 8+16 | 0.702 | 0.672 | 0.687 | 0.37 | 0.699 | 0.681 | 0.690 | 0.38 | 0.001 | 6 | 1 | -0.1 |

**Table S13b:** Performance of various SVM models based on binary pattern & secondary structure; evaluated by non-redundant five-fold cross validation technique using three-way data splits.

| **Window**  **Size** | **validation** | | | | **test** | | | | | | | |
| --- | --- | --- | --- | --- | --- | --- | --- | --- | --- | --- | --- | --- |
|  | Sn | Sp | Ac | Mc | Sn | Sp | Ac | Mc | g | c | j | Th |
| 10+0 | 0.708 | 0.678 | 0.693 | 0.39 | 0.720 | 0.660 | 0.690 | 0.38 | 0.1 | 1 | 3 | 0 |
| 11+0 | 0.751 | 0.663 | 0.707 | 0.41 | 0.705 | 0.635 | 0.670 | 0.34 | 0.01 | 2 | 1 | -0.1 |
| 12+0 | 0.717 | 0.632 | 0.675 | 0.35 | 0.714 | 0.635 | 0.675 | 0.35 | 0.01 | 10 | 6 | 0.2 |
| 0+6 | 0.708 | 0.669 | 0.688 | 0.38 | 0.714 | 0.654 | 0.684 | 0.37 | 0.1 | 1 | 1 | -0.2 |
| 0+7 | 0.711 | 0.726 | 0.719 | 0.44 | 0.672 | 0.705 | 0.688 | 0.38 | 0.1 | 2 | 1 | -0.1 |
| 0+8 | 0.690 | 0.635 | 0.663 | 0.33 | 0.672 | 0.666 | 0.669 | 0.34 | 0.01 | 10 | 1 | -0.2 |
| 6+6 | 0.781 | 0.726 | 0.754 | 0.51 | 0.778 | 0.714 | 0.746 | 0.49 | 0.01 | 4 | 2 | 0.2 |
| 7+6 | 0.815 | 0.693 | 0.754 | 0.51 | 0.806 | 0.678 | 0.742 | 0.49 | 0.01 | 5 | 2 | 0.1 |
| 6+14 | 0.739 | 0.699 | 0.719 | 0.44 | 0.745 | 0.693 | 0.719 | 0.44 | 0.01 | 5 | 2 | 0 |
| 7+14 | 0.726 | 0.708 | 0.717 | 0.43 | 0.714 | 0.726 | 0.720 | 0.44 | 0.01 | 3 | 4 | 0.2 |
| 11+11 | 0.708 | 0.690 | 0.699 | 0.40 | 0.702 | 0.693 | 0.698 | 0.40 | 0.01 | 2 | 5 | 0.3 |
| **6+6+GC** | **0.745** | **0.793** | **0.769** | **0.54** | **0.742** | **0.781** | **0.762** | **0.52** | **0.01** | **5** | **2** | **0.3** |

**Table S14a:** Performance of our method (RISCbinder) at default threshold (0) with highly effective siRNAs data.

| Name | Highly effective siRNA sequences **SS** 5’-------------------------------------3’  **AS** 3’-------------------------------------5’ | **Score** | **Pred** | **Pred#** |
| --- | --- | --- | --- | --- |
| 1. luc-a 2. luc-l 3. luc-k 4. luc-f 5. luc-o 6. luc-5 7. luc-184 8. luc-272 9. luc-309 10. luc-428 11. luc-515 12. luc-658 13. luc-695 14. luc-734 15. luc-774 16. luc-891 17. luc-904 18. luc-1186 19. luc-1308 | ACGCCAAAAACAUAAAGAAAG  UCUGCGGUUUUUGUAUUUCUU  GGUAAAGUUGUUCCAUUUUUU  AGCCAUUUCAACAAGGUAAAA  GGGCGCGGUCGGUAAAGUUGU  GGCCCGCGCCAGCCAUUUCAA  CAUUCUAUCCGCUGGAAGAUG  CGGUAAGAUAGGCGACCUUCU  CCGCCGCCGUUGUUGUUUUGG  AGGGCGGCGGCAACAACAAAA  GACGCCAAAAACAUAAAGAAA  UUCUGCGGUUUUUGUAUUUCU  GUUGGCAGAAGCUAUGAAACG  GCCAACCGUCUUCGAUACUUU  GUGUUGGGCGCGUUAUUUAUC  GCCACAACCCGCGCAAUAAAU  CCGCGAACGACAUUUAUAAUG  CGGGCGCUUGCUGUAAAUAUU  CCAAUCAUCCAAAAAAUUAUU  AGGGUUAGUAGGUUUUUUAAU  CCUCCCGGUUUUAAUGAAUAC  AUGGAGGGCCAAAAUUACUUA  GCAUGCCAGAGAUCCUAUUUU  AGCGUACGGUCUCUAGGAUAA  CCGGAUACUGCGAUUUUAAGU  AAGGCCUAUGACGCUAAAAUU  GGUUUUGGAAUGUUUACUACA  UGCCAAAACCUUACAAAUGAU  GAUUUCGAGUCGUCUUAAUGU  ACCUAAAGCUCAGCAGAAUUA  GCACUCUGAUUGACAAAUACG  UUCGUGAGACUAACUGUUUAU  CAAAUACGAUUUAUCUAAUUU  CUGUUUAUGCUAAAUAGAUUA  GAUUAUGUCCGGUUAUGUAAA  UACUAAUACAGGCCAAUACAU  GCCUGAAGUCUCUGAUUAAGU  GGCGGACUUCAGAGACUAAUU  *(Continued on the following page)* | -1.513  0.621  -0.770  1.062  -0.684  -0.465  -1.842  0.661  -1.636  1.098  -1.187  -0.126  -0.402  0.757  -2.344  0.778  -2.535  2.295  -2.397  1.747  -1.343  0.907  -1.561  0.777  -2.276  2.336  -2.582  1.750  -1.070  0.598  -1.803  1.530  -1.331  0.323  -2.130  0.254  -2.368  1.805 | N  P  N  P  N  N  N  P  N  P  N  N  N  P  N  P  N  P  N  P  N  P  N  P  N  P  N  P  N  P  N  P  N  P  N  P  N  P | N  P  N  P  N  P  N  P  N  P  N  P  N  P  N  P  N  P  N  P  N  P  N  P  N  P  N  P  N  P  N  P  N  P  N  P  N  P |

| Name | Highly effective siRNA sequences **SS** 5’-------------------------------------3’  **AS** 3’-------------------------------------5’ | **Score** | **Pred** | **Pred#** |
| --- | --- | --- | --- | --- |
| 1. luc-1586 2. VIM-270 3. VIM-368 4. VIM-596 5. VIM-812 6. VIM-857 7. VIM-1097 8. VIM-1128 9. VIM-1148 10. VIM-1235   30. VIM-1298  31. Oct-670   1. Oct-797 2. Oct-821 3. EGFP-441 4. EGFP-416 5. DsRed-399 6. DsRed-231 | CUCGACGCAAGAAAAAUCAGA  UUGAGCUGCGUUCUUUUUAGU  CCAUCAACACCGAGUUCAAGA  GCGGUAGUUGUGGCUCAAGUU  CUGGAGCUGCAGAAUAAGAUC  AGGACCUCGUCGUCUUAUUCU  GAAAACACCCUGCAAUCUUUC  GGCUUUUGUGGGACGUUAGAA  GUACGUCAGCAAUAUGAAAGU  UGCAUGCAGUCGUUAUACUUU  GCAGAAGAAUGGUACAAAUCC  UCCGUCUUCUUACCAUGUUUA  GAUGAGAUUCAGAAUAUGAAG  UCCUACUCUAAGUCUUAUACU  CUCGUCACCUUCGUGAAUACC  CCGAGCAGUGGAAGCACUUAU  CAAGACCUGCUCAAUGUUAAG  UGGUUCUGGACGAGUUACAAU  CUGCCUCUUCCAAACUUUUCC  GAGACGGAGAAGGUUUGAAAA  GUUGAUACCCACUCAAAAAGG  ACCAACUAUGGGUGAGUUUUU  GAGAAAGCGAACUAGCAUUGA  UUCUCUUUCGCUUGAUCGUAA  GUUCGAGUAUGGUUCUGUAAC  ACCAAGCUCAUACCAAGACAU  CGCCAGAAGGGCAAAAGAUCA  CCGCGGUCUUCCCGUUUUCUA  GCCACAACGUCUAUAUCAUGG  GUCGGUGUUGCAGAUAUAGUA  CACAAGCUGGAGUACAACUAC  CCGUGUUCGACCUCAUGUUGA  GCCCCGUAAUGCAGAAGAAGA  GCCGGGGCAUUACGUCUUCUU  CCGACAUCCCCGACUACAAGA  GCGGCUGUAGGGGCUGAUGUU | -1.082  0.044  -2.158  1.902  -0.965  0.905  -1.710  -0.399  -1.233  0.934  -0.554  0.374  -0.268  -0.168  -2.041  1.105  -0.881  1.703  -1.595  0.565  -1.162  0.892  -0.109  -1.504  -1.823  0.640  -0.952  0.389  -1.525  0.850  -0.627  -0.266  -2.060  1.504  -2.599  1.691 | N  P  N  P  N  P  N  N  N  P  N  P  N  N  N  P  N  P  N  P  N  P  N  N  N  P  N  P  N  P  N  N  N  P  N  P | N  P  N  P  N  P  N  P  N  P  N  P  N  P  N  P  N  P  N  P  N  P  P  N  N  P  N  P  N  P  N  N  N  P  N  P |

Luc: luciferase, VIM: vimentin, Oct: Oct 4, EGFP: enhanced green fluorescent protein, DsRed: red fluorescent protein, ss: sense strand in 5’🡪3’ direction, AS:antisense strand in 3’🡨5’ direction. Score: classification score of our model, Pred: prediction based on 0 threshold, Pred#: prediction based on relative score of duplex strand, P: positive, N: negative.

**Table S14b:** Performance of our method (RISCbinder) at default threshold (0) with ineffective siRNAs data.

| Name | Highly effective siRNA sequences **SS** 5’-------------------------------------3’  **AS** 3’-------------------------------------5’ | **Score** | **Pred** | **Pred#** |
| --- | --- | --- | --- | --- |
| 1. luc-h 2. luc-m 3. luc-b 4. luc-c 5. luc-14 6. luc-265 7. luc-295 8. luc-411 9. luc-1044 10. VIM-35 11. VIM-155 12. VIM-491 13. Oct-161 14. Oct-566 15. EGCFP-666 16. DsRed-140 17. DsRed-383 | UGGAAGAUGGAACCGCUGGAG  CGACCUUCUACCUUGGCGACC  GGUGAACUUCCCGCCGCCGUU  GGCCACUUGAAGGGCGGCGGC  AAAACAUAAAGAAAGGCCCGG  GUUUUUGUAUUUCUUUCCGGG  UAAAGAAAGGCCCGGCGCCAU  GUAUUUCUUUCCGGGCCGCGG  AACAUAAAGAAAGGCCCGGCG  UUUUGUAUUUCUUUCCGGGCC  UAUGCCGGUGUUGGGCGCGUU  AAAUACGGCCACAACCCGCGC  AGUUGCAGUUGCGCCCGCGAA  CCUCAACGUCAACGCGGGCGC  ACGUGCAAAAAAAGCUCCCAA  CUUGCACGUUUUUUUCGAGGG  UUCUGAUAACACCCGAGGGGG  AUAAGACUAAUGUGGGCUCGC  AGGAUGUUCGGCGGCCCGGGC  CGUCCUACAAGCCGCCGGGCC  UACGCCUCGUCCCCGGGCGGC  AGAUGCGGAGCAGGGGCCCGC  ACCAACGACAAAGCCCGCGUC  AUUGGUUGCUGUUUCGGGCGC  AUCUCCCCAUGUCCGCCCGCA  CCUAGAGGGGUACAGGCGGGC  AUGUGUAAGCUGCGGCCCCUG  UGUACACAUUCGACGCCGGGG  AGUUCGUGACCGCCGCCGGGA  CCUCAAGCACUGGCGGCGGCC  AAGGUGACCAAGGGCGGCCCC  ACUUCCACUGGUUCCCGCCGG  AACUUCCCCUCCGACGGCCCC  ACUUGAAGGGGAGGCUGCCGG | 1.971  -2.264  0.433  -1.006  0.851  -1.780  1.440  -2.760  1.158  -1.611  1.923  -2.375  0.986  -0.742  -0.890  -1.009  1.332  -1.269  -0.793  -2.081  1.010  -2.192  0.023  -0.555  0.358  -1.698  -0.325  -1.829  -0.015  -1.212  0.273  -1.158  -0.186  -1.122 | P  N  P  N  P  N  P  N  P  N  P  N  P  N  N  N  P  N  N  N  P  N  P  N  P  N  N  N  N  N  P  N  N  N | P  N  P  N  P  N  P  N  P  N  P  N  P  N  P  N  P  N  P  N  P  N  P  N  P  N  P  N  P  N  P  N  P  N |

**Table S15:** Features used for various models construction in our study.

.

| **Features** | **Model**  **(Window size)** | **Vector** | **Table** |
| --- | --- | --- | --- |
| Simple nucleotide  composition | Mono | 4 | S2a  S2b |
|  | Di | 16 |  |
|  | Tri | 64 |  |
| Split nucleotide  composition | Mono | 8 | S3 |
|  | Di | 32 |  |
|  | Tri | 128 |  |
| Binary pattern | 6..18 | N x 4 | 1 |
| Binary pattern &  secondary structure | 6..18 | N x 8 | 2 |
| Hybrid of simple  nucleotide composition | Mono+di | 20 | S5 |
|  | Mono+tri | 68 |  |
|  | Di+tri | 80 |  |
|  | Mono+di+tri | 84 |  |
| Hybrid of split  nucleotide composition | Mono+di | 40 |  |
|  | Mono+tri | 136 | S6 |
|  | Di+tri | 160 |  |
|  | Mono+di+tri | 168 |  |
| Hybrid of binary pattern | 6..18+6..18 | Nx4+Nx4 | S7,  S8,S9 |
| Hybrid of binary pattern &  secondary structure | 6..18+6..18 | Nx8+Nx8 | 3,  S10,S11 |
| **Hybrid of binary pattern,**  **secondary structure, & GC** | **6+6+1** | **97** | **RISCbinder** |
| Hybrid of binary pattern,  secondary structure, GC, & Thermodynamics | 6+6+1+1 | 98 | Result section |
